# Application timing and duration of LED and HPS supplements differentially influence yield, nutrient bioaccumulation, and light use efficiency of greenhouse basil across seasons

**DOI:** 10.3389/fpls.2023.1174823

**Published:** 2023-10-31

**Authors:** Hunter A. Hammock, Dean A. Kopsell, Carl E. Sams

**Affiliations:** Department of Plant Sciences, The University of Tennessee, Knoxville, TN, United States

**Keywords:** controlled environment agriculture, light emitting diodes, narrowband LEDs, daily light integral, *Ocimum basilicum*, high pressure sodium, nutrient uptake, supplemental lighting

## Abstract

Three primary factors that impact plant growth and development are light quantity, quality, and duration. Commercial growers can manipulate these parameters using light-emitting diodes (LEDs) to optimize biomass yield and plant quality. There is significant potential to synergize supplemental lighting (SL) parameters with seasonal variation of ambient sunlight to optimize crop light use efficiency (LUE), which could increase biomass while reducing SL electricity costs. To determine the best lighting characteristics and durations for different crops, particularly for enhancing the yield and nutritional quality of high-value specialty crops produced in greenhouses during the winter, a thorough efficacy comparison of progressive incremental daily light integrals (DLIs) using LED and high-pressure sodium (HPS) sources is required. The purpose of this study was to compare the effects of differential application timing and DLIs of supplemental blue (B)/red (R) narrowband wavelengths from LED lighting systems and HPS lamps on greenhouse hydroponic basil *(Ocimum basilicum* var. Genovese) production. We assessed edible biomass, nutrient bioaccumulation, and LUE. Nine light treatments included: one non-supplemented natural light (NL) control, two end-of-day (EOD) HPS treatments applied for 6 h and 12 h, five EOD 20B/80R LED treatments applied for 3 h, 6 h, 9 h, 12 h, 18 h, and one continuous LED treatment (24 h). Each SL treatment provided 100 µmol·m^-2^·s^-1^. The DLI of the NL control averaged 9.9 mol·m^-2^·d^-1^ during the growth period (ranging from 4 to 20 mol·m^-2^·d^-1^). SL treatments and growing seasons significantly impacted biomass and nutrient bioaccumulation; some SL treatments had lower yields than the non-supplemented NL control. January growing season produced the lowest fresh mass (FM) and dry mass (DM) values compared to November, which had the highest. Mineral analyses revealed that both growing seasons and lighting types impacted macro and micronutrient accumulation. Additionally, the efficiency of each treatment in converting electrical energy into biomass varied greatly. EOD supplements using LED and HPS lighting systems both have merits for efficiently optimizing yield and nutrient accumulation in basil; however, biomass and nutrient tissue concentrations highly depend on seasonal variation in ambient sunlight in conjunction with a supplement’s spectral quality, DLI, and application schedule.

## 1 Introduction

Light is one of the most critical factors that impact plant growth, development, and morphology (Briggs and Christie, 2002; Moe et al., 2006). Plants can perceive and respond to a variety of environmental stimuli to ensure survival and reproduction. A sophisticated network of photoreceptors is responsible for sensing and reacting to changes in spectral quality, fluence rate, and duration (Christie, 2007; Yu et al., 2010). Unfavorable environmental conditions can dramatically impact the response of these photoreceptors and prompt other undesirable morphological and physiological responses (Chaves et al., 2011; Fraikin et al., 2013). Specialty crop producers utilize controlled environments and hydroponic cultivation to improve overall yields and quality. Low light intensity and poor spectral quality during winter months force some growers to provide supplemental lighting to achieve successful year-round production and maintain crop quality. Supplemental lighting (SL) systems can satisfy daily light integral (DLI) crop requirements under controlled environments and provide sufficient spectral quality for a variety of greenhouse-grown crops (Fausey et al., 2005; Faust et al., 2005; Currey and Lopez, 2015). In commercial production, growers commonly use high-pressure sodium (HPS) or light-emitting diode (LED) supplements spanning 12-24 h per day, with intensities ranging from 100-200 µmol·m^-2^·s^-1^ for high-value specialty crops (Randall and Lopez, 2014; Singh et al., 2015; Sipos et al., 2020; Engler and Krarti, 2021). HPS lighting systems have traditionally been the predominant choice for commercial operations, but LEDs have many advantages, as well as significant potential for optimizing growth and development characteristics for controlled environment agriculture (Randall and Lopez, 2014).

DLI is defined as the cumulative amount of photosynthetically active photons received by the plant’s canopy in a 24 h period (Fausey et al., 2005). Light requirements vary considerably across species, but it has been generalized that most perennial crops require 10-16 mol·m^-2^·d^-1^ to satisfy quality standards (Faust et al., 2005). Previous studies on a variety of herbaceous crops have revealed that the relationship between DLI and biomass accumulation is mostly linear until approximately 20 or 30 mol·m^-2^·d^-1^ (higher DLIs for a wide range of other species, including C4 crops) (Warner and Erwin, 2003; Faust et al., 2005). Other studies demonstrated linear increases in biomass, plant quality, and inflorescence number on various herbaceous crops with increasing DLI from 5-20 mol·m^-2^·d^-1^ (Fausey et al., 2005; Runkle and Heins, 2006; Frąszczak and Knaflewski, 2009).

The spectral quality of a DLI supplement is a substantial factor. Specific narrowband wavelength supplements can be used to target photoreceptors with the intention of manipulating primary and secondary metabolism, phototropism, and desirable growth/development characteristics (Massa et al., 2008; Carvalho et al., 2016; Wang and Lin, 2020; Santin et al., 2021). Because of their increased energy efficiency, LED lighting systems can significantly reduce the energy costs of commercial producers in the horticulture industry (Engler and Krarti, 2021; Katzin et al., 2021). As electrical grid demand is expected to significantly increase over the upcoming decades, it will be pertinent to conduct further efficacy comparisons between both types of lighting sources along with novel lighting regimes. Additionally, research should be performed to determine the economic feasibly and production quality of LED and HPS lighting systems in relation to non-supplemented greenhouse environments across a wide range of crops, specifically during winter months or unfavorable growing locations (Randall and Lopez, 2014; Ouzounis et al., 2015b; Engler and Krarti, 2021; Katzin et al., 2021).

Studies suggest modern LEDs, particularly systems providing optimized ratios of blue/red wavelengths and broad-spectrum white, are energy efficient and promote desirable morphological characteristics in addition to impacting primary and secondary metabolism, yield, and nutritional value in basil (*Ocimum basilicum*), as well as other specialty crops (Samuolienė et al., 2009; Currey et al., 2012; Kopsell and Sams, 2013; Darko et al., 2014; Currey and Lopez, 2015; Sipos et al., 2021). Additionally, a few recent studies have demonstrated the use of pre-dawn (PD) or end-of-day (EOD) lighting regimes to influence light use efficiency (LUE) and plant metabolism (i.e., circadian rhythm, stomatal conductance, carbon metabolism, nutrient uptake, etc.) with species-specific effects on yield, morphology, and nutrient uptake (Lund et al., 2007; Ouzounis et al., 2015a; Chinchilla et al., 2018; Li et al., 2020; Meng and Runkle, 2020; Vastakaite-Kairiene et al., 2022). It would be advantageous to explore and compare the use of supplemental narrowband LED and HPS treatments, along with natural sunlight, to determine how progressive DLI supplements interact with seasonal variation to spectral quality/DLI in terms of yield and micronutrient tissue concentrations.

The vast majority of higher plants possess natural metabolic cycles, called circadian rhythms, that repeat roughly every 24 h (Harmer, 2009; Venkat and Muneer, 2022). These daily oscillations affect biological processes and are influenced by internal and external cues (Webb, 2003; McClung, 2006). Some aspects of endogenous circadian rhythm can remain under altered environmental conditions for days to weeks, indicating it is generated by a self-sustained central oscillator, which is known as the circadian clock (Hotta et al., 2007; Harmer, 2009; Más and Yanovsky, 2009). The circadian system includes a variety of input routes in addition to the central oscillator that synchronize and entrain it to respond to changes in temperature and light on an hourly, daily, and seasonal basis (Haydon et al., 2013; Venkat and Muneer, 2022). Several physiological and developmental processes that the clock regulates are connected to the central oscillator. The result of this relationship is synchronized physiological and metabolic rhythms with the internal 24 h cycle and the surrounding environment (Más and Yanovsky, 2009; Kim et al., 2017).

Internal and external synchronization cues have varying levels of influence on the endogenous circadian clock, which demonstrates complex signaling channels and mechanisms that regulate circadian synchronization in higher plants. Blue light photoreceptors (cryptochromes) and red/far-red photoreceptors (phytochromes) are known to have a significant role in the synchronization of circadian oscillations to light/dark cycles (Gorton et al., 1993; Millar, 2004). Photosynthesis innately serves as an essential circadian rhythm in plants, composed of diverse metabolic and physiological interactions, including stomatal opening, chlorophyll content, transpiration rate, and net carbon assimilation rate (Harmer et al., 2000; Dodd et al., 2005). For example, one study found that carbohydrates, translocated from shoots to roots, were likely one entraining signal to synchronize circadian oscillations between cells found in roots and shoots (James et al., 2008). Desynchronization of this entraining signal from external cues directly impacts carbon metabolism. External cues with the most potential to impact circadian rhythm physiology are temperature (e.g., changes to daily averages across the growing period, the difference in day/night averages, etc.) and light (e.g., spectral quality, intensity, perceived daylength, DLI), but other environmental stressors will also exert influence (Dodd et al., 2005; Haydon et al., 2013).

To date, there are no published studies that compare narrowband B/R and HPS SL application timing and duration across growing seasons in terms of yield, nutrient uptake, and electrical efficiency. Because horticultural lighting is energy-intensive, it is pertinent to develop lighting strategies to maximize the conversion of SL electrical consumption to biomass. There is significant potential to synergize SL parameters with seasonal variation of ambient sunlight to optimize crop LUE, which could optimize biomass and nutritional quality while reducing SL electricity costs. Due to its high demand and flavor preference, ‘Genovese’ basil makes an excellent model crop to use for light experiments and could predict the impacts of light spectral quality/intensity on other high-value specialty crops (both at the primary and secondary metabolic level), specifically those in the Lamiaceae family (i.e., economically relevant).

We hypothesize that manipulating supplemental lighting quality, application timing, and duration under standard commercial growing conditions has the potential to significantly influence yield, micronutrient bioaccumulation, and LUE of sweet basil across growing seasons. The primary objective of this project was to determine the seasonal impact of application timing and duration of low-intensity EOD SL using specific narrow-band blue (B) and red (R) wavelengths (447 ± 20 nm B and 627 ± 20 nm R) from solid-state LED lighting systems and broadband HPS lamps on edible biomass yield, nutrient bioaccumulation, LUE, and other pertinent energy efficiency metrics of hydroponically grown greenhouse basil. It will be advantageous to establish specific-specific optimal SL protocols (i.e., ideal SL application timing, DLI, intensity, spectral quality, and perceived daylength) that optimize LUE via manipulating crop physiology and circadian rhythms; this provides an opportunity to improve the electrical efficiency of commercial greenhouse SL, while increasing yields and nutrition quality.

## 2 Materials and methods

### 2.1 Experimental site

This present study was conducted at The University of Tennessee Institute of Agriculture (UTIA), which is located in Knoxville, TN, USA (35°56’44.5”N, 83°56’17.3”W). All experiments were conducted under Venlo glass research greenhouses using ebb-and-flow hydroponics. Growing dates for four experimental runs occurred from September 2016 to June 2017. These four experimental runs are labeled as growing seasons.

### 2.2 Experimental design

Emphasis was placed on investigating biomass yield, nutrient bioaccumulation, and LUE in response to differing EOD SL application timing, duration, and spectral quality. This was accomplished using narrowband blue/red (447 nm/627 nm; ± 20 nm) LED light and broadband HPS light applied at equal photosynthetic photon flux densities (PPFD) for different incremental lengths of time across different growing seasons. The 20B/80R LED lighting ratio was specifically chosen for this experiment because of its prevalence in recent literature; it was also deemed optimal for a variety of growth and development parameters from a previous experiment in our group (Hammock et al., 2020).

A total of nine lighting treatments were added immediately after seedling transplantation: one non-supplemented NL control (ambient sunlight only), two HPS treatments applied at 6 h and 12 h per day (Hortilux DE, Mentor, OH, USA), and six 20B/80R LED treatments applied at 3 h, 6 h, 9 h, 12 h, 18 h and 24 h per day (Orbital Technologies, Madison, WI, USA). SL regimes for all treatments were initiated each day 1 h before sunset (except for the 24 h LED treatment, which was continuous after transplantation until day of harvest). Sunset time averages and average daylengths can be found in **Table 1**. Light timers were reset twice weekly to accommodate changing sunset times across growing seasons.

**Table 1:** Environmental parameters (means ± SD) for ‘Genovese’ basil (*Ocimum basilicum* var. Genovese) across distinct growing cycles under greenhouse conditions at The University of Tennessee Institute of Agriculture (UTIA) in Knoxville, TN, USA (35°56’44.5”N, 83°56’17.3”W).

	November	January	March	May
Growing Period	10/14/16-11/20/16	12/28/16-01/29/17	3/10/17-4/24/17	5/01/17-6/12/17
Average Day Temp (°C)	28.9 ± 1.8	27.8 ± 1.2	27.6 ± 1.3	28.0 ± 1.5
Average Night Temp (°C)	22.3 ± 0.5	20.1 ± 0.2	21.8 ± 0.3	22.4 ± 0.5
Average Relative Humidity (%)	55.0 ± 5.0	50 ± 5.0	55 ± 5.0	55 ± 5.0
Average Daily Light Integral (DLI) (mol·m·^-2^·d^-1^)	8.46 ± 3.4	6.89 ± 1.8	9.94 ± 2.1	13.87 ± 3.0
Average Day Length (hours)	9.94	10.02	12.29	14.21
Average Sunset Time (EST)	1739	1747	1840	1948
Average Natural Blue 447 nm (± 5 nm) Intensity at Noon (μmol·m^-2^·s^-1^)	12.7 ± 2.1	11.1 ± 0.4	12.4 ± 1.2	13.8 ± 1.8
Average Natural Red 627 nm (± 5 nm) Intensity at Noon (μmol·m^-2^·s^-1^)	13.3 ± 1.9	11.2 ± 0.9	13.3 ± 1.3	14.5 ± 1.4

**Figure 1:**
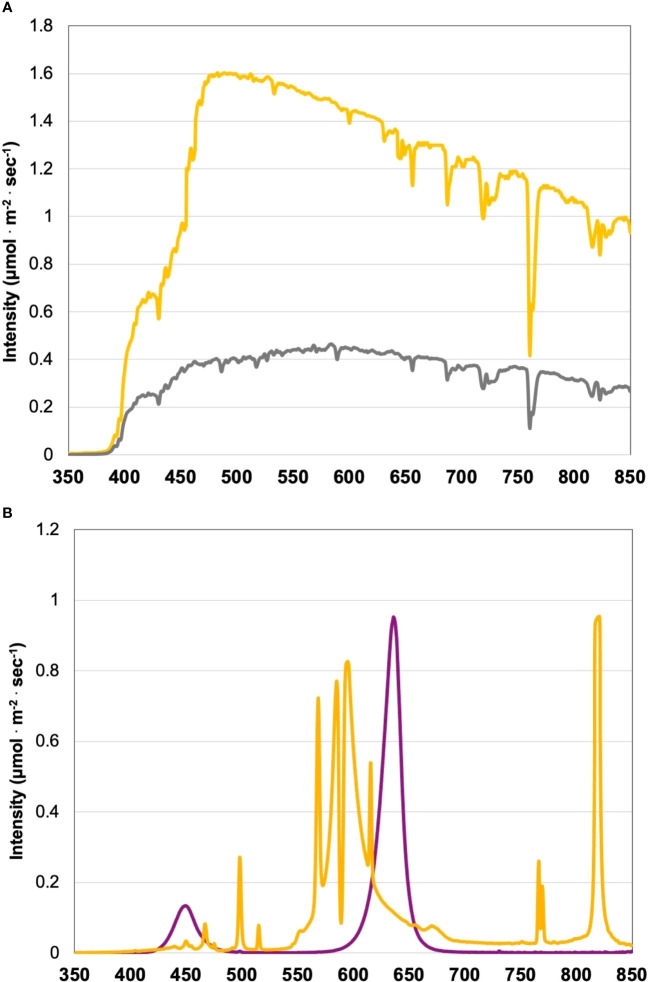
**(A)** Natural light (NL) spectra in greenhouse averaged across all four growing seasons, ranging from 350 nm to 850 nm. Values were taken at solar noon with three replicates for full sun (yellow) and overcast (gray) for each experimental run. The daily light integral (DLI) of the NL control averaged 9.9 mol·m^-2^·d^-1^ across all growing cycles (daily average ranged from 4 to 20 mol·m^-2^·d^-1^ with ± 0.5 mol·m^-2^·d^-1^ variance throughout greenhouse). **(B)** Emission spectra of narrowband LED (purple) and HPS (yellow) treatments from 300 nm to 850 nm. All supplemental lighting (SL) treatments provided 100 ± 2.5 μmol·m^-2^·s^-1^. All lighting treatments were measured with a PS-200 Apogee Spectroradiometer to confirm the intensity of specific treatment wavelengths throughout all growing seasons. Readings were taken after dark in order to exclude underlying natural solar spectra. A total of nine lighting treatments were added immediately after seedling transplant: one non-supplemented natural light control, two HPS treatments with DLIs as 6 h and 12 h, and six 20B/80R LED treatments with progressive DLI as 3 h, 6 h, 9 h, 12 h, 18 h and 24 h.

Each SL treatment uniformly provided 100 ± 2.5 µmol·m^-2^·s^-1^ for its respective duration (uniform intensity and spectral quality distribution verified weekly after dark using average of 5 measurements in a 1 m x 1 m Z pattern across treatment, level with canopy top). LED treatments were 1 m above the hydroponic system, and HPS lamps were 1.5 m above hydroponic systems throughout experiment (post-transplantation). As crops grew, SL intensities were adjusted using dimmers to the target intensity 4-5 times per week (after dark) using a PS-200 Apogee Spectroradiometer (Apogee Instruments, Logan, UT, USA). Leaf temperature variance across treatments was ± 0.4°C from average at canopy surface. The Orbital Technologies LED systems consisted of 10 equally spaced meter-long alternating blue/red bars with adjustable spectral and intensity control, designed to uniformly illuminate 1.2 m x 1.2 m (both spectral quality and intensity). Dimmable HPS lamps were placed in targeted reflectors to reduce SL treatment bleed-over and allow for precise intensity control and uniformity across the 1.2 m x 1.2 m treatment areas. Spectra of lighting treatments and background solar energy have been provided in **Figures 1A, B**.

Each SL treatment was physically separated to ensure no bleed-over effects between SL treatments (average of 1.1 ± 0.6 µmol·m^-2^·s^-1^ SL bleed-over at the treatment edges). 1.2 m x 1.2 m sections of basil were grown, with 1.2 m separation between treatments (i.e., measurement edge-to-edge of hydroponic systems within the greenhouse). Tissue samples for all analyses were only harvested from within the middle 0.6 m x 0.6 m of each treatment to ensure further reduction of SL contamination between treatments (0.3 m around the edge of each treatment was considered the buffer zone and was not used for sampling). Within the harvest zones of adjacent treatments, SL bleed-over was <0.1 µmol·m^-2^·s^-1^ (i.e., below the instrumentation detection limit). Under these circumstances, SL treatment bleed-over was deemed non-significant; therefore, physical barriers (i.e., plastic sheets, boards, etc.) were not utilized because of potential deleterious experimental effects (i.e., interaction with ambient sunlight intensity/DLI, reduced airflow, isolated microclimates leading to air temperature variability, etc.). SL treatments were randomized each season to eliminate the potential for DLI and air temperature variability within the greenhouse bay.

A randomized complete block design was used for this experiment. Lighting treatments were randomized after each experimental cycle to account for seasonal variations in NL intensity, spectral quality, and potential temperature variation within the greenhouse bay. Each measurement unit consisted of two plants to improve statistical power and reduce biological variance; all values and calculations presented have been normalized. Measurement units are presented on a per-plant basis for FM/DMs and nutrient tissue concentrations. Each treatment is considered an experimental unit. Six replicates (two plants each) were analyzed in each treatment (twelve plants, or six measurement units, per treatment within 0.6 m x 0.6 m harvest zone). Each experimental cycle was repeated across four growing seasons (four experimental cycles). Replicates (6) were nested within treatments (9), which were nested within growing seasons (4).

### 2.3 Cultural techniques and growing conditions

Because of its distinctive flavor profile, high market demand, and popularity among chefs, ‘Genovese’ sweet basil was chosen. ‘Genovese’ basil seeds (Johnny’s Select Seeds, Winslow, ME, USA) were germinated in peat moss-based cubes (2 cm × 2 cm × 6 cm; Park’s Bio Dome Sponges, Hodges, SC, USA) under ambient sunlight maintained at 28.3°C and 95% RH. Seeds and sponges were suspended in plastic float trays above 1 L tap water in within humidity domes and trays (Park’s Bio Dome, Hodges, SC, USA).

After 2 weeks in the humidity domes, seedlings were transplanted into plastic pots (8 cm × 8 cm × 9 cm) using 1 part peat moss (Black Gold Canadian Sphagnum Peat Moss, Agawam, MA, USA) to 3 parts perlite (Krum Horticultural Perlite, Hodgkins, IL, USA) potting mix.

Day temperatures averaged 28.1 ± 1.5°C, and night temperatures averaged 21.3 ± 0.4°C. DLI of the natural light control (i.e., ambient sunlight) averaged 9.9 mol·m^-2^·d^-1^ across all four growing seasons (daily average ranging from 4 to 20 mol·m^-2^·d^-1^). Each hydroponic system (treatment) received similar amounts of cumulative ambient sunlight (DLI of ± 0.5 mol·m^-2^·d^-1^ across treatments) throughout the four growing seasons, in addition to the prescribed SL regime. Relative humidity during the growth period averaged 55%.

Specific growing parameters for each of the seasons may be found in **Table 1**, which were collected using greenhouse control sensors (PRIVA, Ontario, CA), WatchDog 2000 Series sensors (Spectrum Technologies, Aurora, IL, USA), and PS-200 Apogee Spectroradiometer (Apogee Instruments, Logan, UT, USA).

All basil plants were grown in ebb-and-flow hydroponic systems and sub-irrigated for 5 min each day with full-strength general mix nutrient solution; the fertility regime was kept constant across the duration of all seasons. Total growth time lasted approximately 45 d across all 4 experimental runs (seasons). The nutrient solution was kept consistent at 5.9 ± 0.1 pH and 2.0 ± 0.1 dS/m, changed at least weekly. Nutrient solution consistent of a modified Hoagland solution using fertilizer grade mineral salts. Each bench (three treatments) had its own closed nutrient reservoir.

Nutrient solution samples were analyzed throughout each experiment to ensure consistent nutrient composition across reservoirs and seasons. Water samples were taken twice weekly in 15 mL sterile test tubes. A 9.9 mL of acid matrix solution (2% nitric acid, 0.5% hydrochloric acid, 97.5% RO water) was placed into 15 mL sterile test tubes. A disposable 1 mL plastic pipette was used to add 0.1 mL of the homogenized nutrient solution sample to the 9.9 mL acid matrix solution. This mixture was then thoroughly shaken to ensure that the nutrient solution sample was uniformly distributed within the matrix. An Agilent 7500 Series Inductively Coupled Plasma Mass Spectrometer (ICP-MS) was used to determine the concentrations of each nutrient solution sample (Barickman et al., 2013). Average concentrations measured in nutrient solutions were as follows (ppm): N (207.54), P (50.87), K (298.23), Ca (180.15), Mg (77.10), S (136.45), Fe (3.95), Mn (0.90), Zn (0.40), Mo (0.09), Cu (0.90), and B (0.90).

### 2.4 Sampling and data collection

**Figure 2:**
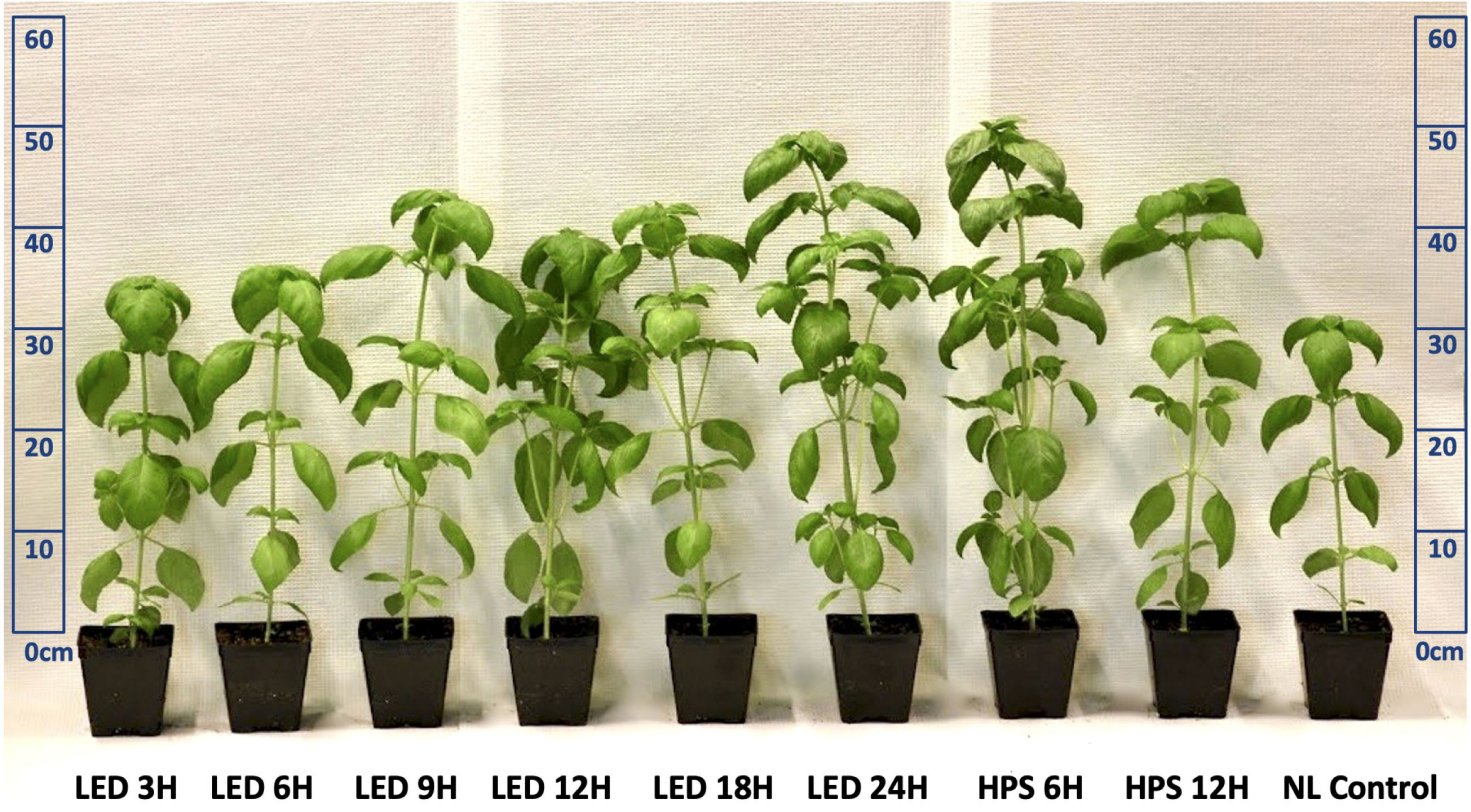
Visual representation of LED lighting impacts on morphology of hydroponically grown ‘Genovese’ basil (*Ocimum basilicum* var. Genovese). Photo taken immediately before May season harvest and shows comparison of variations in height, canopy size, leaf area, and pigmentation after being exposed to supplemental blue (B)/red (R) LEDs and broad-spectrum HPS. A non-supplemented natural NL control was used to account for daily light integral (DLI) and spectral quality variations across seasons.

Plants were harvested at physiological vegetative maturity (approximately 45 days after seeding, with 9-10 fully developed nodes) for each growing season. **Figure 2** shows representative morphological and developmental changes imparted by SL treatments. All tissue samples were collected by replication within the same four-hour period. Biomass samples were collected within the 0.6 m x 0.6 m harvest zone (two plants per measurement unit, six replicates per treatment). A total of 108 plants were harvested across all treatments and replicates per season. Fresh mass (FM) and dry mass (DM) were collected using an analytical scale (Sartorius L310, Sartorius, Göttingen, Germany). Tissue samples were air dried for 128 h at 50 °C using a forced air dryer (Heratherm OMH100 Drying Oven, Thermo Scientific, Waltham, MA, USA). Samples were immediately weighed and processed after removing from drying oven.

### 2.5 Analytical procedures

To determine changes in elemental nutrient tissue concentrations in basil plants across SL treatments and growing seasons, samples were analyzed for macro and micronutrient concentrations according to a method from Barickman et al. (2013). Air-dried samples were ground into a fine powder using a Magic Bullet blender (MBR1101, Homeland Housewares, Los Angeles, CA, USA). 0.5 ± 0.01 g of ground plant material was weighed into 15 mL sterile plastic centrifuge test tubes. An Ethos 1112 microwave digestion unit (Milestone, Bergamo, Italy) was used to process the basil samples. Samples were microwaved for 30 min at 150 °C, then cooled for an additional 30 min. A 9.9 mL of ICP matrix solution (2% nitric acid, 0.5% hydrochloric acid, 97.5% RO water) was placed into 15 mL sterile test tubes. A disposable 1 mL plastic pipette was used to add 0.1 mL of the acid-digested sample mixture to the 9.9 mL ICP matrix solution. This mixture was then thoroughly shaken to ensure that the acid was uniformly distributed within the matrix. An Agilent 7500 Series Inductively Coupled Plasma Mass Spectrometer (ICP-MS) was used to determine the nutrient concentrations of each tissue sample (Barickman et al., 2013). Using this method, elemental tissue analysis provides sample concentrations of P, K, Ca, S, Mg, B, Cu, Mn, Fe, Na, and Zn.

### 2.6 Calculation of energy efficiency

Energy efficiency and cost efficacy calculations of EOD SL were evaluated for hydroponically grown greenhouse basil. Efficiency values are calculated on both a per-plant FM basis (harvested within the buffer zone with six replicates per treatment, two plants per measurement unit, averaged across all four seasons) and a full system FM basis (entire FM of each respective lighting treatment/hydroponic system, averaged across growing seasons, to accurately determine the influence of SL energy on the total yield of each cropping system at commercial capacity). LUE was calculated on a per-plant DM basis. All calculations (except LUE) are based on statistical averages across all four growing seasons intended to determine generalized efficiency parameters representative of a year-round growing operation under standard greenhouse conditions; LUE is evaluated by treatment and growing season individually.

The calculations in this study only consider the cost of energy consumed by lighting fixtures; calculations do not include other greenhouse sources of energy consumption, service charges from electricity providers, lighting fixture purchase, or maintenance, which are all important factors when deciding SL types and regimes.

First, daily SL electrical energy input (kWh·d^-1^) was calculated based on the time in hours (h) each treatment was provided and the electrical requirement of the energy fixture in Watts (W).


(1) $$\frac{kW*h}{d}=kWh\cdot d^{‐1}$$

Next, the total SL electrical energy input per harvest cycle (kWh_T_) was calculated by multiplying the daily electrical input (kWh·d^-1^) per treatment by the number of days (d) per growing cycle.


(2) $$kWh*d^{-1}*d=\;kWh_{T}$$

The total SL energy cost per growing cycle (USD_T_) was then determined based on the current electrical rate in Knoxville, TN. Electrical energy was provided to our growing facility by Knoxville Utility Board (KUB) at the current rate of $0.10 kWh (USD per kWh).


(3) $$kWh_{T}*\frac{0.10\;USD}{kWh}=USD_{T}$$

The DLI provided by each SL treatment per day (DLI_SL_) and the total perceived DLI (i.e., per day average of treatment, DLI_SL_, plus per day average DLI of natural light, DLI_NL_; DLI_T_) were calculated using SL treatment instantaneous photosynthetic photon flux density (PPFD) of 100 µmol·m^-2^·s^-1^, treatment duration s_TD_, and known average DLI received inside the greenhouse.


(4) $$\left[100\;mol*m^{-2}*s^{-1}\right]*[s_{SL}]*[\frac{1\;mol}{1000000\;mol}]*[d^{-1}]=mol*m^{-2}*d^{-1}\;DLI_{SL}$$

and


(5) $$DLI_{SL}+DLI_{NL}=DLI_{T}$$

The average perceived daylength (h_T_) was also calculated for each treatment, which was the total amount of time within a 24 h period that a treatment received light (all values averaged across seasons). One hour is subtracted from h_SL_ because treatments were initiated one hour prior to sunset. Maximum h_T_ is equal to 24 h (i.e., h_T_ ≤ 24 h).


(6) $$h_{NL}+\left(h_{SL}-1h\right)=h_{T}$$

Biomass efficiency (BE) is a measure of the conversion of light energy by a crop into biomass. Per plant biomass efficiency (*ɡ*·DLI^-1^) was calculated based on the yield and total DLI average received by each treatment.


(7) $$\frac{ɡ}{DLI_{T}}=BE$$

Average per plant yield increase over NL control (*ɡ*_PPY_) was calculated, which is useful for comparing the impact (positive or negative) of SL treatments with the non-supplemented control. It is the yield difference (*ɡ*) of the non-supplemented NL control (*ɡ*_NL_) and each SL treatment (*ɡ*_SL_).


(8) $${ɡ}_{SL}-{ɡ}_{NL}={ɡ}_{PPY}$$

Average total system yield increase over NL control (*ɡ*_TSY_) was also calculated, which is the fresh mass difference (*ɡ*) of the total FM of the non-supplemented NL control (*ɡ*_TNL_) and total FM of each SL treatment (*ɡ*_TSL_). This value provides a reference point to determine the efficiency of each SL treatment’s ability to convert electrical energy to FM increases over the NL control with respect to the entire 1.2 m x 1.2 m hydroponic system.


(9) $${ɡ}_{TSL}-{ɡ}_{TNL}={ɡ}_{TSY}$$

The SL energy input per total system yield increase ( $\text{kW}{\text{h}}_{\text{T}}\cdot {ɡ}_{\text{TSY}}^{\;-1}$ ) was calculated for each treatment, which shows the amount of electrical energy (kWh_T_) required per g increase of total system FM (i.e., across all 49 plants under each treatment) increase over the NL control (*ɡ*_TSY_).


(10) $$\frac{kWh_{T}}{{ɡ}_{TSY}}=kWh_{T}*{ɡ}_{\text{TSY}}^{\;-1}$$

Positive $\text{kW}{\text{h}}_{\text{T}}\cdot {\text{ɡ}}_{\text{TSY}}^{\;-1}$ values represent electrical energy being utilized to increase total system yield over the NL control; a small positive value indicates a more efficient conversion of electrical energy to biomass, while a larger positive value indicates the lighting source is less electrically efficient for increasing total system yield. Negative values represent electrical energy being used from SL to detrimentally influence total system yield as compared to the NL control; a small negative value (i.e., relative to zero, absolute value) indicates a significant reduction in biomass per unit of electricity, while an increasing negative value (i.e., relative to zero, absolute value) indicates the lighting treatment had less impact on a reduction in total system yield. A small positive value would be considered ideal in terms of the conversation of electrical energy to total system FM increase over the NL control.

To quantify in dollars, the total system yield increase per SL energy cost ( ${ɡ}_{\text{TSY}}\cdot USD_{T}^{\;-1}$ ) was calculated, which is the amount of yield difference (*ɡ*_TSY_) per dollar in SL electrical energy (USD_T_).


(11) $$\frac{{ɡ}_{TSY}}{USD_{T}}={ɡ}_{TSY}*USD_{T}^{\;-1}$$

While this value will vary based on current electrical rates, location, and currency, it can be used to put a relative dollar value on the SL regime comparison, as well as the cost efficacy of SL treatments for increasing FM over non-supplemented ambient sunlight. Positive values represent the amount of yield increase expected per dollar of energy input; the larger the value, the higher the cost-effectiveness of the lighting treatment for increasing FM over the NL control. Negative values represent the amount of yield decrease expected per dollar of energy input increase; more negative values (i.e., relative to zero, absolute value) indicate higher yield losses per USD of electricity spent. Large positive values are ideal, which indicates high-cost efficacy in terms of energy conversion to FM increase over NL control.

Finally, light use efficiencies (LUE, g of dry weight per mol of incident light throughout life cycle) were calculated for each lighting treatment ( ${\text{LUE}}_{\text{T}};{ɡ}_{\text{DM}}\cdot \text{mo}{\text{l}}_{T}^{\;-1}$ ) and season ( ${\text{LUE}}_{\text{S}};{ɡ}_{\text{DM}}\cdot \text{mo}{\text{l}}_{S}^{\;-1}$ ).


(12) $$\frac{{ɡ}_{DM}}{mol_{SL}+mol_{NL}}=LUE_{T}$$

And


(13) $$\frac{{ɡ}_{DM}}{mol_{S}}=LUE_{S}$$

LUE is a physiological measure that indicates a plant’s ability to convert light energy into chemical energy, and will vary based on species and environmental conditions. Evaluating energy efficiency parameters and LUE across treatments will provide insight on the optimization of SL application timing, duration, and spectral quality for the yield of sweet basil.

### 2.7 Statistical Analysis

Data sets were analyzed by GLIMMIX and Mixed Model Analysis of Variance procedures using the statistical software SAS (version 9.4, SAS Institute, Cary, NC, USA). Design and Analysis macro (DandA.sas; created by Arnold Saxton) was utilized in addition to Tukey’s (protected) adjustment, regression analysis, and univariate/normalization procedures. Treatments and seasons were separated by least significant difference (LSD) at α=0.05.

## 3 Results

Fresh mass (FM) and dry mass (DM), as well as the nutrient concentrations of edible tissue, were evaluated in this experiment. Data presented include plant weights (g), mineral concentrations (macronutrients in mg·g^-1^ DM and micronutrients in µg·g^-1^ DM), and energy efficiency calculations.

### 3.1 Biomass

**Figure 3:**
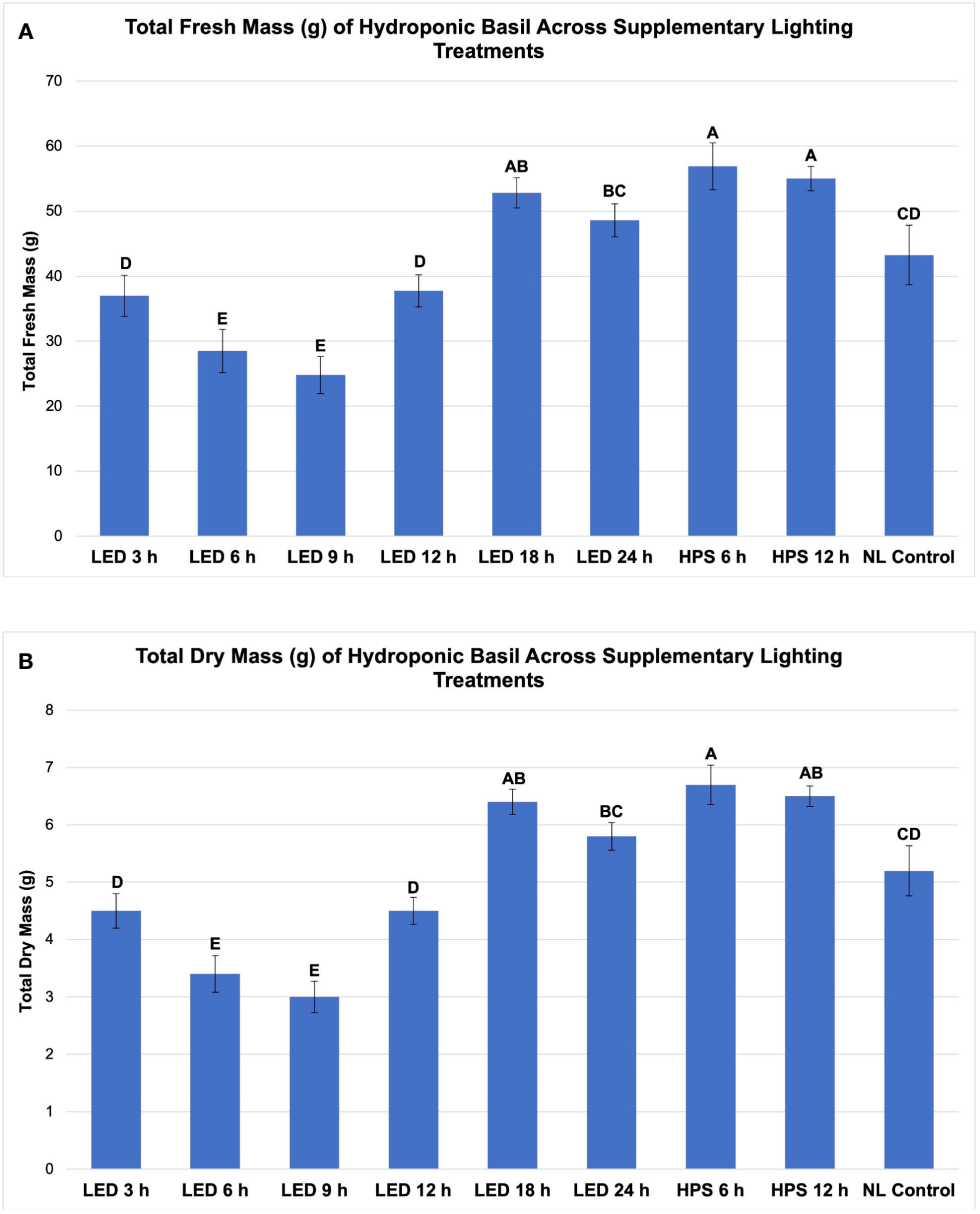
Influence of progressive DLIs increments on **(A)** total plant fresh mass and **(B)** total plan dry mass of hydroponically grown 'Genovese' basil (Ocimum basilicum var. Genovese). A total of nine lighting treatments were added immediately after seedling transplant: one non-supplemented natural light (NL) control, two HPS treatments with DLIs as 6 h and 12 h, and six 20B/80R LED treatments with progressive DLI as 3 h, 6 h, 9 h, 12 h, 18 h and 24 h. All weights are presented in grams (g). Mean values represent 2 plants per replication and 6 replications per treatment. Values were analyzed using Tukey's (protected) LSD, and those followed by the same letter are not significantly different (α=0.05). Error bars represent SD.

**Figure 4:**
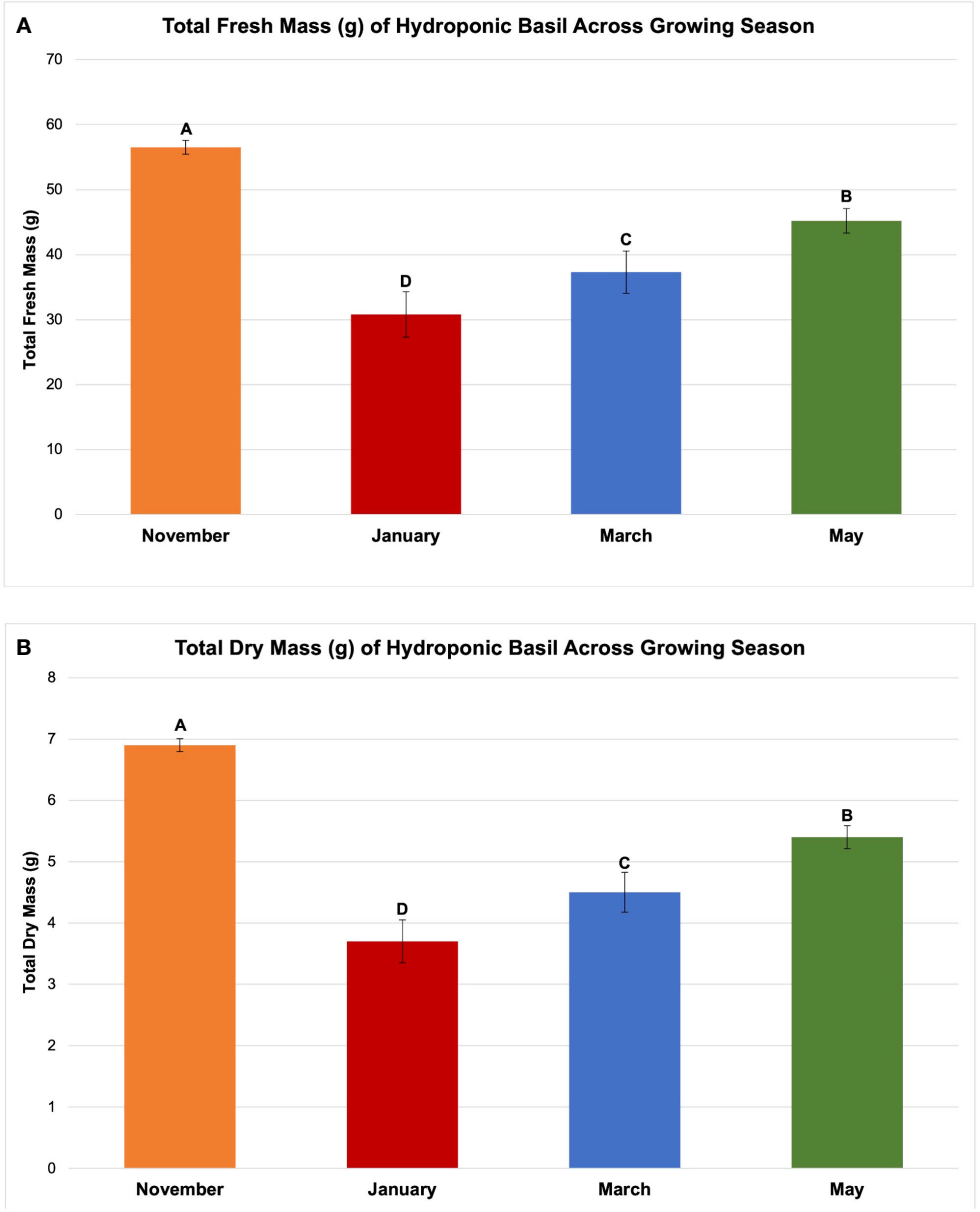
Influence of growing season on **(A)** total plant fresh mass and **(B)** total plant dry mass of hydroponically grown 'Genovese' basil (Ocimum basilicum var. Genovese). All weights are presented in grams (g). Mean values represent 2 plants per replication and 6 replications per treatment across each season. Values were analyzed using Tukey's (protected) LSD, and those followed by the same letter are not significantly different (α=0.05). Error bars represent SD.

**Figure 5:**
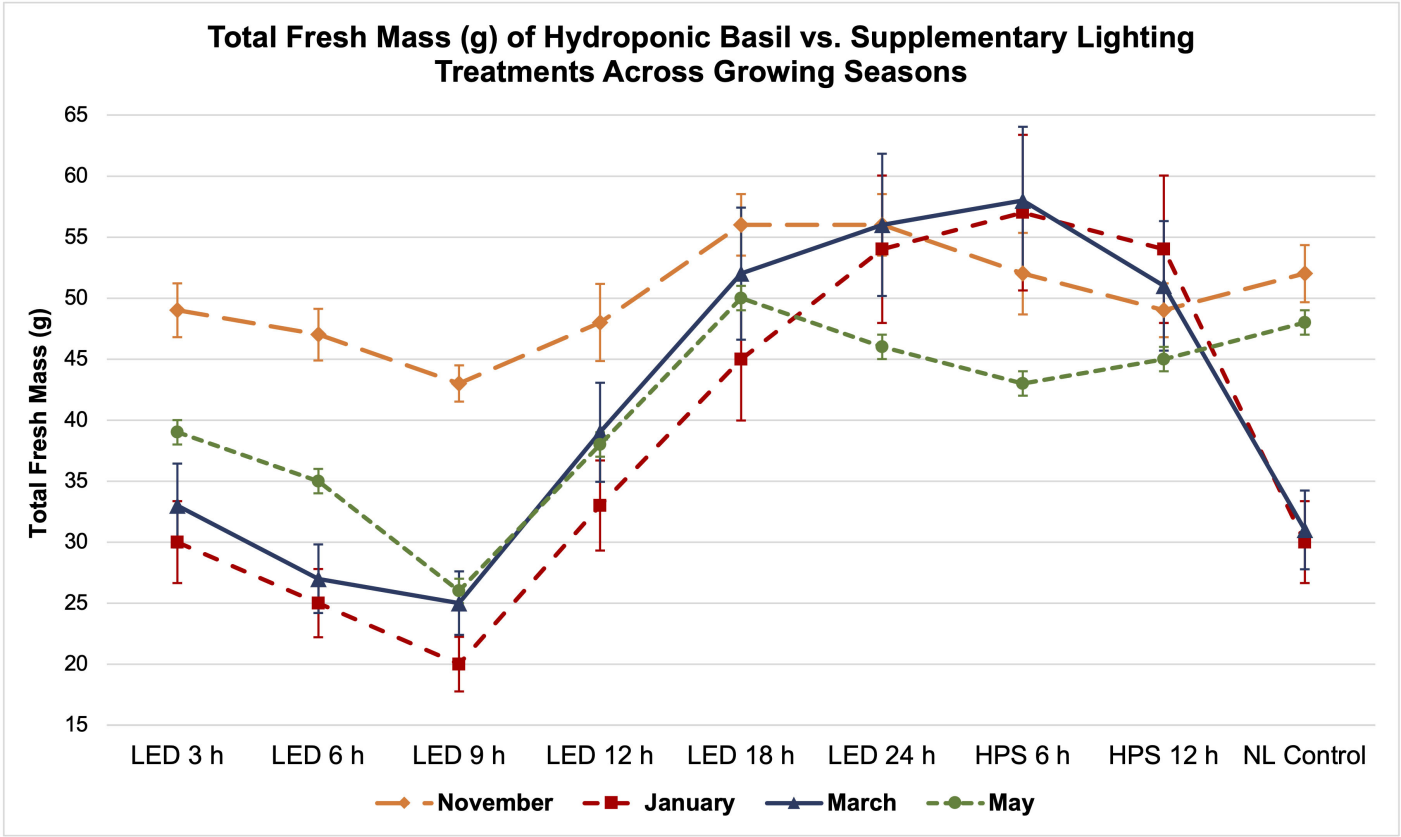
Influence of DLIs increments on total plant fresh mass of hydroponically grown ‘Genovese’ basil (*Ocimum basilicum* var. Genovese) across seasons. A total of nine lighting treatments were used: one non-supplemented natural light (NL) control, two HPS treatments with DLIs as 6 h and 12 h, and six 20B/80R LED treatments with progressive DLI as 3 h, 6 h, 9 h, 12 h, 18 h and 24 h. Four growing cycles were conducted during the months of November, January, March, and May. All weights are presented in grams (g). Mean values represent 2 plants per replication and 6 replications per treatment. Error bars represent SD.

**Figure 6:**
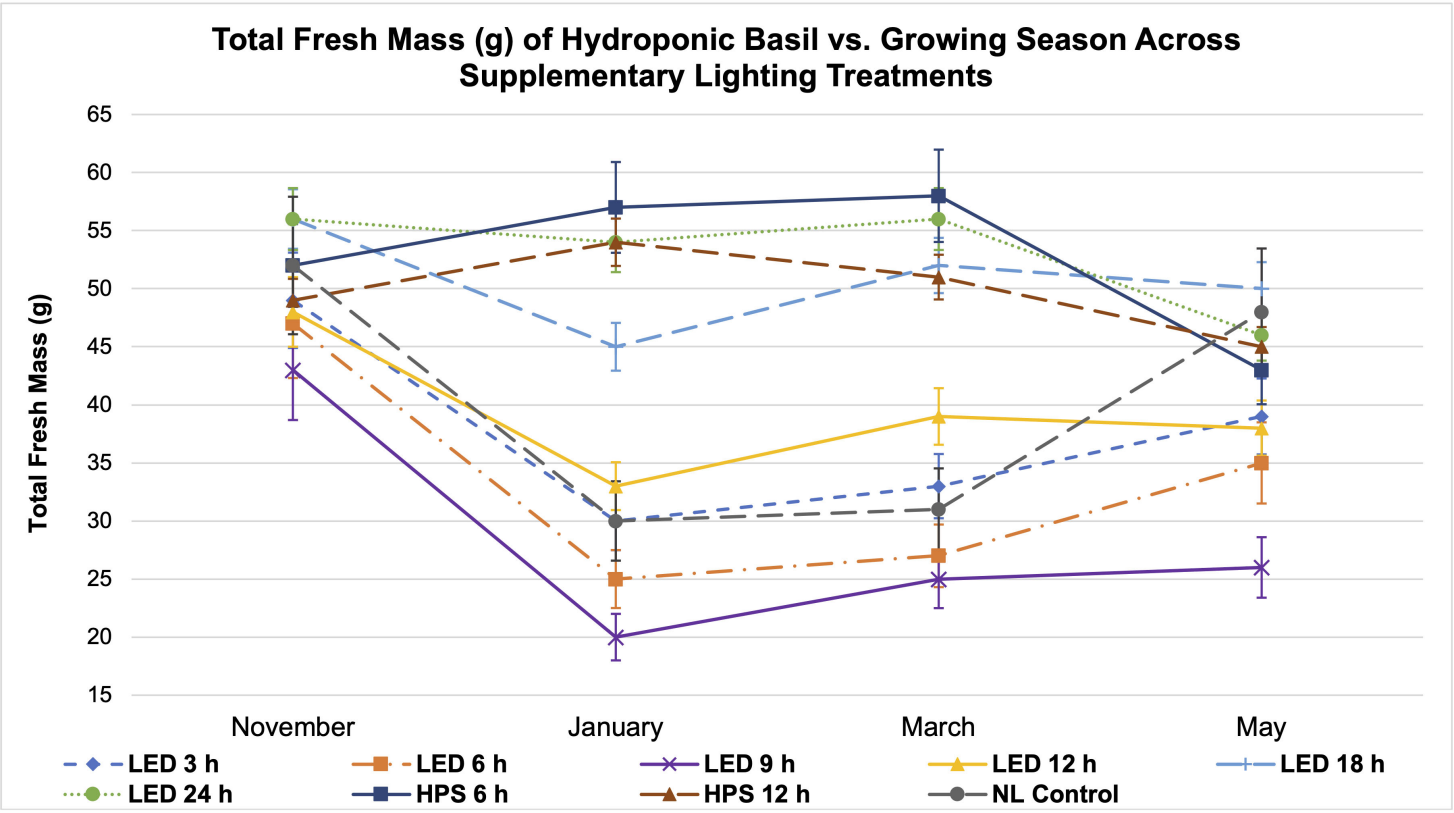
Influence of season on total plant fresh mass of hydroponically grown ‘Genovese’ basil (*Ocimum basilicum* var. Genovese) across lighting treatments. A total of nine lighting treatments were used: one non-supplemented natural light (NL) control, two HPS treatments with DLIs as 6 h and 12 h, and six 20B/80R LED treatments with progressive DLI as 3 h, 6 h, 9 h, 12 h, 18 h and 24 h. Four growing cycles were conducted during the months of November, January, March, and May. All weights are presented in grams (g). Mean values represent 2 plants per replication and 6 replications per treatment across growing seasons. Error bars represent SD.

As expected, FM and DM were significantly impacted across growing seasons and lighting treatments; patterns that resulted from inherent characteristics of each lighting system, SL treatment (amount of progressive DLI increment), spectral quality variations, and natural DLI provided across growing seasons will be discussed (**Figures 3A, B**, **4A, B**, **5**, **6**).

Total FM was significantly impacted by lighting treatment (*P ≤* 0.0001; F=62.23) and season (*P ≤* 0.0001; F=138.36) but did not show significant season*treatment interactions (*P*=0.1235; F=9.62). When comparing yield across LED treatments, a clear sigmoidal pattern is observed. HPS lighting treatments generally performed better than LED treatments. The 6 h and 12 h HPS treatments had higher FM than most of the LED treatments, showing a 27% increase over the NL control (**Figure 3A**). The optimal LED treatment was 18 h and did not statistically separate from the HPS treatments; it was only 3 g less than the 6 h HPS treatment. The optimal LED treatment averaged 53 g FM per plant. The NL control averaged 43 g, which was higher than some of the narrowband SL treatments. Despite having higher DLIs than the NL control, the 6 h and 9 h LED treatments had the statistically lowest FM, between 25 and 30 g. This was approximately a 30% reduction in yield from the NL control (**Figure 3A**).

Total plant DM followed a similar pattern to FM and was significantly impacted by lighting treatment (*P ≤* 0.0001; F=61.21) and season (*P ≤* 0.0001; F=144.24) but not by season*treatment interactions (*P*=0.2651; F=7.88). HPS lighting treatments 6 h and 12 h again produced the two highest DMs across all growing seasons, averaging 6.8 g and 5.9 g DM per plant, respectively (**Figure 3B**). The optimal LED treatment was 18 h and did not statistically separate from the HPS treatments; it was only 0.2 g less on average than the 6 h HPS treatment. The NL control produced 5.2 g DM in comparison to the optimal LED treatment, which was 6.4 g. The best HPS and LED treatments increased DM over NL control by approximately 25-29%. The lowest DM was again produced under the 6 and 9 h LED treatment, which was 3.0 g (43% decrease from NL control) and 3.4 g (35% decrease from NL control), respectively (**Figure 3B**).

The November growing season produced the highest FM and DM, while the lowest was produced in the January growing season (**Figures 4A, B**). November produced 58 g FM on average, as compared to January, with 31 g FM. DM/FM ratio was evaluated across treatments and seasons, but no statistically significant separations were found in the present study.

### 3.2 Nutrient composition

Tissue P was significantly impacted by lighting treatment (*P*=0.0035; F=3.00) and season (*P ≤* 0.0001; F=47.30) but did not show season*treatment interactions (*P*=0.1051; F=1.42). There were elevated levels of P in the 12 h LED treatment and decreased levels in the 6 h LED treatment; however, most of the treatments did not statistically separate, except for the two previously mentioned (**Table 2**). The January and May seasons accumulated the most P in comparison to the March growing season. January had a 46% increase in P tissue concentrations over March, and May had an approximately 40% increase over March (**Table 3**). Tissue K was significantly impacted by season (*P ≤* 0.0001; F=8.82) and season*treatment interactions (*P*=0.0043; F=2.05) but not by lighting treatment (*P*=0.0531; F=2.21). Plants grown in January had significantly higher K concentrations, while none of the other seasons were statistically different (**Table 3**). Tissue Ca was not significantly impacted by lighting treatment (*P*=0.0636; F=1.89), season (*P*=0.0768; F=2.32), or season*treatment interactions (*P*=0.1134; F=1.40) (**Tables 2**, **3**). Tissue S was significantly impacted by lighting treatment (*P ≤* 0.0001; F=5.91), season (*P ≤* 0.0001; F=57.33), and season*treatment interactions (*P ≤* 0.0001; F=2.95). The highest treatment tissue concentrations of S were 24 h LED and 6 h HPS, while the lowest treatment was 6 h LED (**Table 2**). Plants grown in January and May had significantly higher S concentrations, while November had the lowest S concentrations (**Table 3**). Tissue Mg was significantly impacted by lighting treatment (*P*=0.0118; F=2.25), season (*P ≤* 0.0001; F=11.78), and season*treatment interactions (*P*=0.0283; F=1.70). Mg concentrations in the 18 h LED treatment and the 6 h HPS treatment were found to be higher in comparison to the 6 h LED treatment; however, there was variation across treatments, and many did not statistically separate (**Table 2**). November had the highest tissue concentrations of Mg, while March had the lowest, and the other seasons did not statistically separate (**Table 3**).

**Table 2:** Influence of supplemental lighting treatments on macronutrient mineral concentrations of hydroponically grown ‘Genovese’ basil (*Ocimum basilicum* var. Genovese).

Light Treatment	P (mg·g^-1^)	K (mg·g^-1^)	Ca (mg·g^-1^)	S (mg·g^-1^)	Mg (mg·g^-1^)
LED 3 h	8.47 ± 1.98^abc^	53.71 ± 8.42^a^	16.45 ± 2.21^ab^	4.48 ± 1.29^bc^	7.02 ± 1.02^ab^
LED 6 h	7.50 ± 1.66^c^	49.83 ± 10.36^a^	15.74 ± 2.48^b^	4.16 ± 1.01^c^	6.54 ± 1.03^b^
LED 9 h	8.33 ± 1.65^abc^	53.03 ± 8.62^a^	17.88 ± 2.73^ab^	4.98 ± 1.27^abc^	7.12 ± 1.22^ab^
LED 12 h	8.99 ± 2.23^a^	48.97 ± 5.67^a^	18.37 ± 2.74^a^	5.37 ± 1.82^ab^	7.58 ± 1.07^ab^
LED 18 h	8.90 ± 2.76^ab^	48.81 ± 11.89^a^	17.09 ± 3.44^ab^	4.97 ± 1.49^abc^	7.72 ± 1.89^a^
LED 24 h	7.99 ± 1.82^abc^	47.15 ± 7.50^a^	16.92 ± 2.76^ab^	5.55 ± 1.68^a^	7.55 ± 1.28^ab^
HPS 6 h	7.84 ± 1.62^abc^	45.98 ± 8.13^a^	17.47 ± 2.46^ab^	5.63 ± 1.13^a^	7.71 ± 1.46^a^
HPS 12 h	7.85 ± 2.14^abc^	49.67 ± 12.33^a^	17.43 ± 3.21^ab^	4.73 ± 1.20^abc^	7.34 ± 1.33^ab^
NL Control	7.59 ± 2.01^bc^	46.49 ± 12.97^a^	16.43 ± 4.17^ab^	4.63 ± 1.68^bc^	6.91 ± 1.84^ab^

*All concentrations are presented in milligrams per gram dry plant weight (mg·g^-1^ DM). Mean values represent 2 plants per replication and 6 replications per treatment. Values were analyzed using Tukey’s (protected) LSD, and those followed by the same letter are not significantly different (α=0.05). A total of nine light treatments were added immediately after seedling transplant: one non-supplemented natural light (NL) control, two HPS treatments as 6 h and 12 h, and six 20B/80R LED treatments as 3 h, 6 h, 9 h, 12 h, 18 h and 24 h.

**Table 3:** Influence of growing season on macronutrient mineral concentrations of hydroponically grown ‘Genovese’ basil (*Ocimum basilicum* var. Genovese).

Growing Season	P (mg·g^-1^)	K (mg·g^-1^)	Ca (mg·g^-1^)	S (mg·g^-1^)	Mg (mg·g^-1^)
November	7.40 ± 1.35^b^	48.98 ± 8.14^b^	16.19 ± 2.91^a^	3.73 ± 0.78^c^	7.91 ± 1.52^a^
January	9.57 ± 2.12^a^	53.88 ± 13.21^a^	17.39 ± 3.75^a^	6.01 ± 1.72^a^	7.06 ± 1.35^bc^
March	6.53 ± 1.01^c^	45.53 ± 6.27^b^	17.31 ± 1.85^a^	4.46 ± 0.55^b^	6.58 ± 0.61^c^
May	9.16 ± 1.83^a^	48.78 ± 9.31^b^	17.44 ± 3.10^a^	5.57 ± 1.34^a^	7.56 ± 1.40^ab^

*All concentrations are presented in milligrams per gram dry plant weight (mg·g^-1^ DM). Mean values represent 2 plants per replication and 6 replications per treatment, which is nested within four seasons. Values were analyzed using Tukey’s (protected) LSD, and those followed by the same letter are not significantly different (α=0.05).

Tissue B was significantly impacted by lighting treatment (*P ≤* 0.0001; F=5.32), season (*P ≤* 0.0001; F=62.03), and season*treatment interactions (*P*=0.0027; F=2.14). The 9h LED treatment had the highest B concentrations across all growing seasons, while the lowest was observed under the 6 h LED treatment (**Table 4**). Plants grown in January had significantly higher B concentrations in comparison to all other seasons, with November and May being statistically lowest (**Table 5**). Tissue Cu was significantly impacted by lighting treatment (*P*=0.0040; F=2.96), season (*P ≤* 0.0001; F=37.24), and season*treatment interactions (*P*=0.0019; F=2.20). The lowest Cu concentrations were found in the NL control and 24 h LED (**Table 4**). Plants grown in November had the highest concentrations, and the lowest concentrations were found in January and May (**Table 5**). Tissue Mn was significantly impacted by lighting treatment (*P*=0.0359; F=2.12), season (*P ≤* 0.0001; F=37.62), and season*treatment interactions (*P*=0.0024; F=2.16). 12 h LED had higher concentrations than 6 h LED, but the other treatments did not statistically separate (**Table 4**). Plants grown in January showed the highest Mn concentrations, while all other months were significantly lower (**Table 5**). Tissue Fe was significantly impacted by lighting treatment (*P*=0.0005; F=3.74), season (*P ≤* 0.0001; F=42.11), and season*treatment interactions (*P*=0.0116; F=1.87). 9 h LED had statistically greater tissue concentrations of Fe as compared to the 12 h HPS and NL control, while the other treatments did not separate (**Table 4**). Plants grown in January had significantly higher Fe concentrations, while the November growing season had the lowest concentrations; March and May did not statistically separate (**Table 5**). Tissue Na was significantly impacted by lighting treatment (*P*=0.0002; F=4.13), season (*P ≤* 0.0001; F=84.89), and season*treatment interactions (*P ≤* 0.0001; F=3.48). The 6 h LED treatment had the highest concentrations, while the lowest was found in 6 h HPS. The NL control fell between the Na tissue concentrations range for this experiment (**Table 4**). Plants grown in the November season had significantly higher Na concentrations, while spring seasons had lower Na concentrations (**Table 5**). Tissue Zn was significantly impacted by lighting treatment (*P*=0.0106; F=3.74) and season (*P ≤* 0.0001; F=7.21), but no significant season*treatment interactions (*P*=0.1026; F=1.42) were observed. The 9 h LED treatment and NL control were the only two Zn tissue concentrations that statistically separated (**Table 4**). Plants grown in November had significantly higher Zn concentrations, while the lowest was observed in the May season; January and March seasons did not statistically separate (**Table 5**).

**Table 4:** Influence of supplemental lighting treatments on micronutrient mineral concentrations of hydroponically grown ‘Genovese’ basil (*Ocimum basilicum* var. Genovese).

Light Treatment	B (µg·g^-1^)	Cu (µg·g^-1^)	Mn (µg·g^-1^)	Fe (µg·g^-1^)	Na (µg·g^-1^)	Zn (µg·g^-1^)
LED 3 h	73.86 ± 12.01^bc^	30.65 ± 12.12^a^	112.4 ± 19.29^ab^	179.8 ± 31.68^ab^	246.6 ± 83.73^abc^	56.77 ± 10.70^ab^
LED 6 h	64.54 ± 14.71^c^	28.11 ± 15.85^a^	104.1 ± 24.49^b^	168.6 ± 38.70^abc^	278.4 ± 95.12^a^	54.66 ± 8.94^ab^
LED 9 h	74.90 ± 15.54^bc^	28.41 ± 12.35^a^	117.4 ± 22.46^ab^	187.4 ± 41.37^a^	258.6 ± 72.47^ab^	60.02 ± 6.95^a^
LED 12 h	89.29 ± 25.16^a^	29.95 ± 13.53^a^	127.1 ± 35.21^a^	173.8 ± 48.43^abc^	214.8 ± 73.92^bc^	57.52 ± 6.13^ab^
LED 18 h	80.45 ± 25.77^ab^	24.71 ± 9.93^ab^	117.4 ± 35.17^ab^	164.7 ± 45.11^abc^	237.1 ± 74.84^abc^	54.32 ± 11.35^ab^
LED 24 h	76.36 ± 19.24^bc^	21.84 ± 7.89^b^	110.2 ± 25.21^ab^	169.4 ± 38.21^abc^	228.4 ± 66.39^bc^	53.33 ± 7.37^ab^
HPS 6 h	74.46 ± 20.07^bc^	22.91 ± 10.84^ab^	117.2 ± 22.44^ab^	164.8 ± 27.73^abc^	205.3 ± 83.23^c^	53.53 ± 6.96^ab^
HPS 12 h	75.91 ± 18.38^bc^	22.66 ± 7.84^ab^	113.1 ± 27.77^ab^	153.3 ± 44.79^bc^	232.5 ± 69.75^abc^	52.89 ± 9.59^ab^
NL Control	77.95 ± 20.52^ab^	21.09 ± 8.92^b^	110.5 ± 31.54^ab^	143.9 ± 44.89^c^	236.6 ± 85.22^abc^	51.32 ± 11.58^b^

*All concentrations are presented in micrograms per gram dry plant weight (µg·g^-1^ DM). Mean values represent 2 plants per replication and 6 replications per treatment. Values were analyzed using Tukey’s (protected) LSD, and those followed by the same letter are not significantly different (α=0.05). A total of nine light treatments were added immediately after seedling transplant: one non-supplemented natural light (NL) control, two HPS treatments as 6 h and 12 h, and six 20B/80R LED treatments as 3 h, 6 h, 9 h, 12 h, 18 h and 24 h.

**Table 5:** Influence of growing season on micronutrient mineral concentrations of hydroponically grown ‘Genovese’ basil (*Ocimum basilicum* var. Genovese).

Growing Season	B (µg·g^-1^)	Cu (µg·g^-1^)	Mn (µg·g^-1^)	Fe (µg·g^-1^)	Na (µg·g^-1^)	Zn (µg·g^-1^)
November	67.91 ± 11.91^c^	36.11 ± 4.21^a^	107.3 ± 19.27^b^	141.2 ± 33.41^c^	325.1 ± 41.21^a^	58.48 ± 8.59^a^
January	97.70 ± 21.05^a^	19.98 ± 7.08^c^	141.2 ± 34.36^a^	208.9 ± 46.71^a^	251.1 ± 39.91^b^	56.44 ± 10.31^ab^
March	75.04 ± 12.97^b^	29.31 ± 11.94^b^	102.8 ± 14.27^b^	163.2 ± 28.93^b^	175.8 ± 42.71^d^	52.67 ± 7.43^bc^
May	65.01 ± 12.29^c^	16.95 ± 2.96^c^	106.1 ± 20.06^b^	155.8 ± 33.45^bc^	198.4 ± 43.80^c^	52.11 ± 8.72^c^

*All concentrations are presented in micrograms per gram dry plant weight (µg·g^-1^ DM). Mean values represent 2 plants per replication and 6 replications per treatment, which is nested within 4 seasons. Values were analyzed using Tukey’s (protected) LSD, and those followed by the same letter are not significantly different (α=0.05).

### 3.3 Energy efficiency

An energy analysis revealed that application timing, duration, and spectral quality of SL influenced a number of energy efficiency and cost parameters (**Tables 6**, **7**). The biomass efficiency across treatments varied over two-fold, from 1.89 g·DLI^-1^ to 4.72 g·DLI^-1^; the HPS and NL control had higher biomass efficiencies, while the lowest was found under the LED treatments. The lowest was under the LED 9 h treatment, which illustrates the importance of both DLI and SL application timing; adding energy in the form of 9 h LED reduced the biomass efficiency by 56% compared to the NL control (**Table 7**).

**Table 6:** Energy consumption and general parameters of supplementary lighting (SL) on hydroponically grown ‘Genovese’ basil (*Ocimum basilicum* var. Genovese).

Light Treatment	SL Energy Input (Watts)	SL Energy Input Per Day (kWh·d^-1^)	SL Total Energy Input Per Harvest Cycle (kWh)	SL Energy Total Cost (USD)*	Supplemented DLI (mol·m^-2^·d^-1^)**	Total Treatment DLI (mol·m^-2^·d^-1^)**	Total Daylength (h)**
LED 3 h	150.00	0.45	20.25	2.03	1.08	10.98	13.65
LED 6 h	150.00	0.90	40.50	4.05	2.16	12.06	16.65
LED 9 h	150.00	1.35	60.75	6.08	3.24	13.14	19.65
LED 12 h	150.00	1.80	81.00	8.10	4.32	14.22	22.65
LED 18 h	150.00	2.70	121.5	12.15	6.48	16.38	24.00
LED 24 h	150.00	3.60	162.00	16.20	8.64	18.54	24.00
HPS 6 h	1000.00	6.00	270.00	27.00	2.16	12.06	16.65
HPS 12 h	1000.00	12.00	540.00	54.00	4.32	14.22	22.65
NL Control	0.00	0.00	0.00	0.00	0.00	9.90	11.65

*Electricity assumed $0.10 per kWh, which is the current Knoxville, TN rate.

**Average across all four growing seasons, as perceived by crop.

**Table 7:** Efficacy comparison of supplementary lighting (SL) on hydroponically grown ‘Genovese’ basil (*Ocimum basilicum* var. Genovese) in terms of energy consumption and fresh biomass increase.

Light Treatment	Average Plant Fresh Yield (g)*	Average Total System Fresh Yield (g)**	Per Plant Biomass Efficiency (g·DLI^-1^)*	Average Plant Yield Increase Over NL Control (g)*	Average Total System Yield Increase Over NL Control (g)**	SL Energy Input per Total System Yield Increase (kWh·g^-1^)**	Total System Yield Increase per SL Energy Cost (g·USD^-1^)**
LED 3 h	37.1	1820	3.38	-6.20	-310.0	-0.06	-152.70
LED 6 h	28.5	1390	2.36	-14.80	-740.0	-0.05	-182.70
LED 9 h	24.8	1220	1.89	-18.50	-910.0	-0.07	-149.60
LED 12 h	37.7	1850	2.65	-5.60	-280.0	-0.28	-34.60
LED 18 h	52.8	2590	3.22	9.50	460.0	0.26	37.90
LED 24 h	48.6	2380	2.62	5.30	250.0	0.64	15.50
HPS 6 h	56.9	2790	4.72	13.60	660.0	0.40	24.50
HPS 12 h	55.2	2710	3.88	11.90	580.0	0.93	10.70
NL Control	43.3	2130	4.37	0.00	0.0	–	–

*Grams per plant basis, average across four growing seasons.

**Average across all four growing seasons.

The average plant yield increase over NL control (g_PPY_) compares all treatment FMs to the NL control FM, which allows the ability to incorporate positive/negative values for later calculations. LED 18 h, 24 h, and HPS 6 h and 12 h had increased yields over the NL control, while other treatments had reduced yields. Average plant yield increase over NL control ranged from 13.60 g (HPS 6 h) to -18.50 g (LED 9 h) (**Table 7**).

The average total system yield increase over NL control (g_TSY_) is related to the average plant yield (g_PPY_) and represents the total FM (positive or negative) yield as compared to the NL control. This value is necessary for total-system energy efficiency calculations. The LED 18 h, 24 h, and HPS 6 h and 12 h treatments had increased over the NL control, while other LED treatments had reduced yields. Average total system yield increase over NL control ranged from 660.0 g (HPS 6 h) to -910.0 g (LED 9 h) (**Table 7**). In other words, the application of some SL treatments improved total system yield, while others reduced it.

SL energy input per total system yield increase (kWh_T_·ɡ_TSY_^-1^) represents the amount of SL electrical energy required per gram of total system FM increase over the NL control (**Table 7**). Values range from 0.93 kWh·g^-1^ (HPS 12 h) to -0.28 kWh·g^-1^ (LED 12 h). Half of the treatments (LED 3-12 h) were negative, indicating a decrease in the total system yield below the NL control (despite paying for SL electricity), while the other half (LED 18-24 and HPS 6-12) increased total system yield and various rates (with each additional kWh_T_).

To quantify SL electricity usage in dollars, the total system yield increase per SL energy cost (g·USD^-1^) was calculated (**Table 7**). This value can be used to compare the cost efficacy of SL treatments for increasing FM over non-supplemented ambient sunlight. Values of total yield increase per SL energy cost range from 37.90 g·USD^-1^ (LED 18 h) to -182.70 g·USD^-1^ (LED 6 h). In other words, the LED 18 h treatment was the most cost-effective in terms of increasing total system yield, while the LED 6 h treatment was the least cost-effective treatment and actually led to significant losses in total system yield.

**Figure 7:**
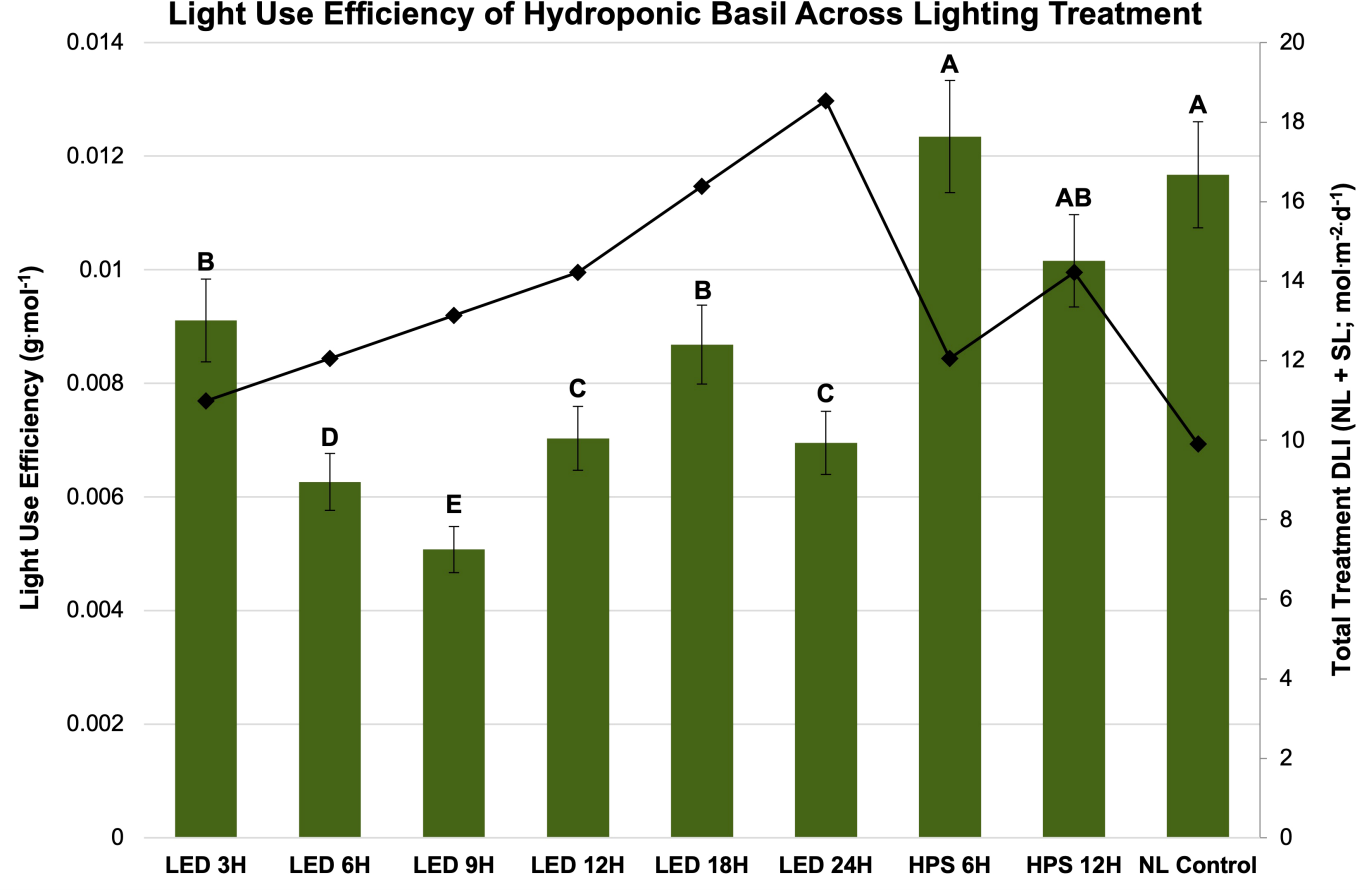
Influence of lighting treatments on light use efficiency (LUE; g·mol^-1^) of hydroponically grown ‘Genovese’ basil (*Ocimum basilicum* var. Genovese) across lighting treatments. Calculated total treatment DLI (NL + SL) is shown on the secondary axis for reference. A total of nine lighting treatments were used: one non-supplemented natural light (NL) control, two HPS treatments applied for 6 h and 12 h, and six 20B/80R LED treatments applied for 3 h, 6 h, 9 h, 12 h, 18 h, and 24 h. Values were analyzed using Tukey’s (protected) LSD, and those followed by the same letter are not significantly different (α=0.05). Error bars represent SD.

**Figure 8:**
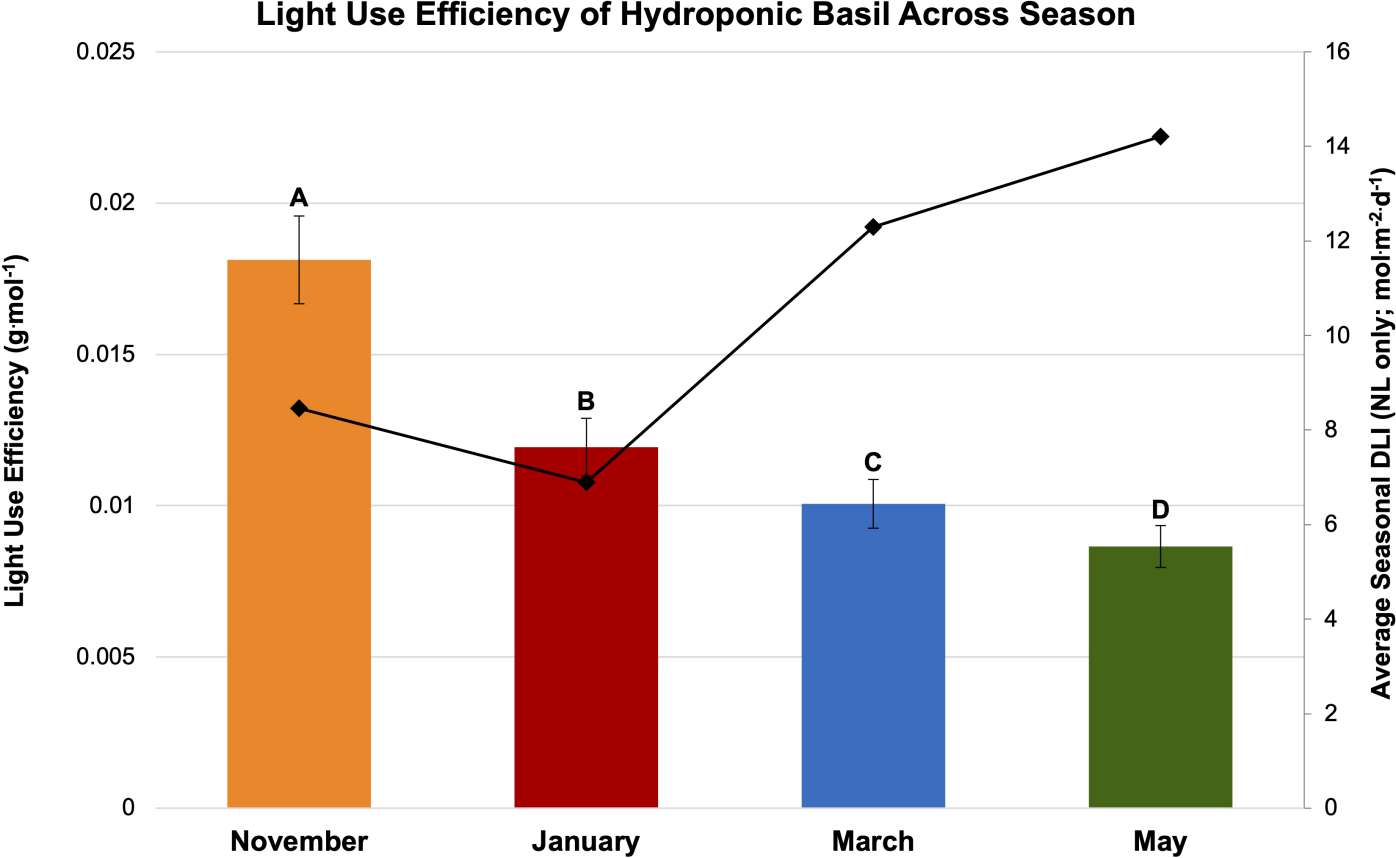
Influence of season on light use efficiency (LUE; g·mol^-1^) of hydroponically grown ‘Genovese’ basil (*Ocimum basilicum* var. Genovese). Calculated seasonal average DLI is shown on the secondary axis for reference. Seasons include November, January, March, and May. Values were analyzed using Tukey’s (protected) LSD, and those followed by the same letter are not significantly different (α=0.05). Error bars represent SD.

Finally, LUE was calculated for SL treatment (**Figure 7**) and season (**Figure 8**). LUE is a physiological measure of how well a plant utilizes available photosynthetically active photons during its life cycle. LUE values were significantly influenced by treatment (*P ≤* 0.0001; F=6.01) and season (*P ≤* 0.0001; F=3.32). LUE was statistically highest for the HPS 6 h, HPS 12 h, and NL control. The many of lowest values were found under the LED treatments between 6 h and 24 h (**Figure 7**). November had the highest LUE, while May had the lowest. All the seasonal LUEs statistically separated (**Figure 8**).

## 4 Discussion

Supplementing natural sunlight with EOD LED and HPS treatments across growing seasons in greenhouse hydroponic production allows for the manipulation and comparison of three key lighting parameters: spectral quality, DLI, and perceived daylength. Exploring the interaction of these parameters on primary metabolism allows the optimization of lighting treatments for yield, nutritional value, and energy efficiency.

### 4.1 Influence of daily light integral on biomass

Spectral quality, DLI, and perceived daylength from SL had an influence on basil FM and DM. Interestingly, the 6 h HPS lighting treatment produced the highest total biomass (fresh and dry) across all growing seasons evaluated. It was significantly higher than the NL control and many of the LED treatments (**Figures 3A, B**). Despite receiving 2x SL DLI, the 24 h LED treatment yielded significantly less than the 12 h HPS treatment. The NL control achieved higher FM and DM than four of the six LED treatments, which was unexpected.

It has generally been understood that within a species-specific range, DLI is linearly correlated with crop yield. For example, Marcelis et al. (2006) found that, as a rule of thumb, a 1% light increment results in a 0.5% to 1% increase in harvestable product for most crops. Baumbauer et al. (2019) stated that increasing DLI positively influenced the fresh weight (FW) of leaf lettuce (*Lactuca sativa)*. Other experiments have shown that increased DLI is correlated with increased FM and DM (Warrington and Norton, 1991; Faust et al., 2005; Gent, 2014; Malik and Heród, 2022). In this experiment, we did not observe a direct linear correlation between increased DLI supplement and basil biomass (**Table 1**; **Figures 5**, **6**). January consistently had the lowest DLI and lowest average yields. November had the highest average yields, despite May having the highest average DLI of any other growing season. The yield averages of all LED treatments were higher during November as compared to other growing seasons (**Figure 5**). Decreasing daylength and increasing red wavelengths within the ambient solar spectrum during November may have caused the narrowband treatments to be more effective moving towards harvest date, as compared to the increasing daylength and blue wavelengths within the ambient solar spectrum during the May season. While DLI (natural sunlight, SL, and specifically the cumulative total) clearly influenced yield in this study, the results further suggest the interactions between SL treatment, season, and NL DLI are responsible for differences in yield. Keeping photosystems active 24 h per day without oversaturating them may be helpful for optimizing FM while saving energy costs (assuming species is tolerant of 24 h photoperiod); conversely, sub-optimal DLI, insufficient spectral quality, and inadequate photoperiod schedules can reduce biomass accumulation in a variety of plant species (Goins et al., 1997; Briggs and Huala, 1999; Moe et al., 2006; Runkle and Heins, 2006).

Two notable patterns emerge when comparing seasonal yield across treatments (**Figure 5**). First, lighting type had different performance based on season. The HPS treatments generally performed better in winter months, while the LED supplements performed better during November and May (higher DLI than winter months). Since the SL treatments were initiated 1 h before sunset, a boost of red/FR wavelengths in addition to the extra radiant heat energy from HPS lighting systems during winter nights may have increased photosynthetic rates and kept light-dependent reactions active throughout the night period, without significantly impacting endogenous circadian rhythm. HPS treatment yields during November and May were diminished as compared to winter months, likely from the same excess radiant heat during night hours (**Figure 5**). The LED SL treatments did not possess this excess radiant heat but did provide specific narrowband wavelengths known to induce transcriptome and metabolic changes, which likely led to the variation in yields across seasons. This is clearer in **Figure 6**, where the two HPS treatments make an “arch shape” across seasons (i.e., higher yields for HPS during winter, lower in higher DLI months), while the LED treatments and NL control generally followed a “bowl shape” across seasons (i.e., higher yields moderately trended with seasonal DLI increases, where the November and May yields were relatively higher than the winter months).

Second, natural DLI significantly influenced the amount of impact across seasonal treatments (**Figures 5**, **6**). November, which had the second highest DLI, had the least amount of variation in FM across treatments; January, which had the lowest DLI of any season, had the widest range across treatments (i.e., 9 h LED treatment vs. 6h HPS) (**Figure 5**). The higher the DLI, the less influence SL treatments had on yield. November growing season averages in the 3 h to 9 h LED treatments were also dramatically higher when compared to other seasons (**Figure 5**). This may be explained by previously discussed factors that have significant impacts on plant growth and development, including photomorphogenic responses, variation in spectral quality across growing seasons, thermal differences between HPS/LED treatments, and effects of spectral quality on photosynthetic and respiration rates.

Because the DLI supplements were applied 1 h before sunset, primary metabolic rates may have been significantly impacted as photosystems remained fully active for some of the optimal treatments after sunset. Based on daylength calculations, the 18 h LED treatment resulted in a perceived ‘24 h photoperiod’ for the basil crop (**Table 6**). 24 h LED treatment may have provided above-optimal additions of B/R wavelengths that negatively impacted biomass production in comparison to the 18 h LED treatment. The 9 h LED treatment had the lowest yields across all seasons, likely because it received a brief dark period before sunrise, affecting circadian rhythm, photosynthetic machinery, and other metabolic processes responsible for assimilating carbon and mineral elements. One study found that 500 μmol·m^-2^·s^-1^ for 16 h per day produced optimal edible biomass production for sweet basil (*Basilicum ocimum* L.) in a controlled environment production system (Beaman et al., 2009). It is generally well established that oversaturation (of intensity or cumulative DLI) is detrimental to yield (Faust et al., 2005). The 24 h LED treatment also provided an additional 2.1 mol·d^-1^ of B/R wavelengths during the morning hours as compared to the 18 h treatment, which shut off around sunrise. This suggests that spectral quality and SL schedule optimization are essential factors to consider when maximizing yield, in addition to the total DLI received by a crop. DLI is a crucial factor in greenhouse production, but the choice of lighting type and spectral quality will significantly impact biomass yields, all of which are highly dependent on the growing season and specific greenhouse crop.

We speculate that the unexpected variation observed across progressive DLI supplements is a result of the interaction between seasonal ambient sunlight and SL spectral quality, application timing, and DLI provided, by influencing essential metabolic functions such as circadian rhythm, stomatal conductance, carbon metabolism, and nutrient acquisition. To fully understand the impact that each SL treatment had on primary metabolism, it is important to compare the differences across treatments and seasons in terms of not only DLI but also specific temporal application of discrete wavebands and perceived daylength in relation to the endogenous circadian clock of basil plants.

Over 30% of primary metabolite accumulation of *Arabidopsis* is under circadian control, which regulates numerous enzymes that are involved with primary metabolism (Webb, 2003; McClung, 2006; Haydon et al., 2013). Other studies have demonstrated circadian rhythm regulating metabolic pathways linked to carbon fixation and allocation between starch and sucrose located in leaf tissue (Harmer et al., 2000; Wang and Lin, 2020; Li et al., 2021). During the day, photosynthesis leads to the fixation of CO_2_ into starch molecules, which are then stored until night (Graf and Smith, 2011; Stitt and Zeeman, 2012). At night, these molecules are broken down and used as an energy source until daybreak. However, when the onset of night is unexpected or manipulated, it can result in an early depletion of the starch reserves and cause carbon starvation. This has been shown to have a major impact on metabolic pathways and gene expression, which can ultimately lead to noticeable alterations in biomass production within a period of 2-3 weeks (Zeeman et al., 2010; Stitt and Zeeman, 2012; Kim et al., 2017). There is also evidence to suggest that synchronization between endogenous circadian rhythm and environmental conditions enhances photosynthesis and carbohydrate metabolism (Dodd et al., 2005; Hotta et al., 2007; Zeeman et al., 2010; Graf and Smith, 2011; Stitt and Zeeman, 2012).

### 4.2 Influence of spectral quality on biomass

The two types of lighting used in this experiment, LEDs and HPS, have vastly different emission spectra (**Figure 1B**). The LED treatments in this experiment have two primary narrowband wavelengths. These include 447 nm and 627 nm (± 20 nm each) at 20B/80R. The LED treatments do not have any other wavebands in their emission spectra. On the other hand, the HPS treatments have multiple peaks across the visible spectrum (**Figure 1B**). There are very small peaks around 450 nm and 475 nm. There is a significant peak at 500 nm. The majority of the HPS spectra are comprised of wavelengths from 550 nm to 650 nm. There is another significant peak at 770 nm, which is past the PAR spectrum and approaching infrared (IR). HPS also had a significant peak at 825 nm, which will be given off as radiant heat (IR wavelengths). These two types of lighting were applied EOD, which impacts perceived spectral quality across each treatment’s perceived daylength. The lighting treatments in this experiment provided sole source lighting at night and, when applicable, manipulated the solar spectrum provided during the day (based on time of day and season). Each LED treatment provided the same spectral quality and intensity, with progressively increasing DLIs. The HPS lights had the same intensity as the LED treatments, but the spectral quality of light they provided differed from the LEDs (**Figure 1B**).

To further investigate spectral quality’s impact on yield, we can directly compare the 6 h and 12 h LED treatments to the 6 h and 12 h HPS treatments; each respective treatment type applied the same intensity and duration of SL, with different spectral quality. Despite providing the same amount of SL (i.e., intensity and DLI), the 6 h LED treatment had 53% less yield than the 6 h HPS treatment. The 12 h LED treatment had 69% less yield than the 12 h HPS treatment. Increasing SL duration from 6 h to 12 h significantly increased the yield for the LED treatments, but it did not significantly change the yield between the HPS treatments. Additionally, the 6 and 12 LED treatments did not improve yields over the NL control (**Figure 3A**). There are three notable differences in spectral quality between the lighting systems: 1) the LED peak at 450 nm vs. the HPS peak at 500 nm; 2) the LED peak 630 nm vs. the HPS peaks between 550-625 nm; and 3) the HPS peak around 770 nm, which is absent on the LED. Spectral variance within these three ranges will impact the response of cryptochromes and phytochromes, directly influencing circadian rhythm and other metabolic processes.

Cryptochromes (CRYs) are blue light pigment-proteins (photoreceptors) that regulate light-mediated plant growth and development (Ahmad, 2016). CRYs are responsible for various blue light responses of plants, which include circadian clock synchronization and entrainment (Somers et al., 1998; Yanovsky et al., 2001; Lin and Todo, 2005), inhibition of hypocotyl elongation (Ahmad and Cashmore, 1993; Ma et al., 2016), stimulation of stomatal opening (Briggs and Huala, 1999; Mao et al., 2005; Christie, 2007; Li et al., 2021), carbon metabolism (Li et al., 2021; Venkat and Muneer, 2022), and other light-dependent stress responses (Chaves et al., 2011; Ahmad, 2016; Wang and Lin, 2020). The absorption spectrum for CRYs has sharp peaks around 420 nm, then sharply drops off around 500 nm (Malhotra et al., 1995; Ahmad, 2016); the LED lighting system provides enough blue intensity (447 nm) within the action spectra of CRYs to stimulate a response, while the HPS has significantly less blue wavelengths. Phytochromes (PHYs) work in conjunction with CRYs and other photoreceptors to elicit transcriptomic, metabolic, and photomorphogenic responses (Casal, 2007). These photoreceptors perceive minute variations in spectral quality, DLI, and daylength, which is a crucial input signal for day/night entrainment with the endogenous plant circadian oscillator (Somers et al., 1998; Millar, 2004).

Specific responses in this study could be related to contradictory information from photoreceptors, in which signals from red light PHYs indicated the incidence of light, but the lack of blue light can be interpreted as darkness. In *Arabidopsis*, a suite of five photoreceptor classes provides the ability to perceive minor variations in spectral quality ranging from ~280 nm to 780 nm (Briggs and Huala, 1999; Mas et al., 2000; Briggs and Christie, 2002; Lin and Todo, 2005; Liu et al., 2013). A recent meta-analysis revealed that spectral sensitivity is more complex than previously thought and suggests green light should be divided between shortwave and longwave responses, with shorter wavelengths of green light acting to complement blue light-induced responses, whereas longer wavelengths antagonize blue light signaling events, either through the direct repression of CRY signaling or via a PHY-dependent mechanism (Battle et al., 2020).

There is a slight but significant spectral shift in the two lighting types in this experiment, between the ranges of 550-650 nm; this range had most of the SL intensity compared to other wavelengths across the spectrum. Additionally, the HPS treatment had a peak of around 770 nm. We speculate the difference in yields between the comparable 6 h LED and 6 h HPS treatment differentially influenced primary metabolism via CRY/PHY signal-mediated responses. The 12 h HPS provided red and FR wavelengths prior to sunrise, which may have been responsible for the lack of linear increase of yield with DLI. It is likely that the varying spectral quality between HPS and LED lighting treatments used in this experiment differentially targeted CRY and PHY photoreceptors, leading to a range of primary metabolic responses. Further, these treatments were applied EOD, some of which would conflict with endogenous circadian rhythm inputs regarding the day/night entrainment, producing cascading and profound impacts on both carbon metabolism and source/sink signaling. Additional experiments are needed to verify this concept.

### 4.3 Potential interactions influencing biomass and nutrient bioaccumulation

Interactions between light treatment and growing season significantly impacted many of the nutrient concentrations evaluated, namely elevated macronutrient concentrations found in 12-24 h LED treatments during the January growing season. This likely occurred because plants grown in January had lower FM averages in general, while the 12-24 h LED treatments in this season resulted in some of the highest biomasses for that growing period. Because of the increased biomass, additional macronutrient concentrations are needed to satisfy nutrient requirements and facilitate healthy plant growth and development. Relatively low macro and micronutrient concentrations were observed in the NL control, especially in the January growing season.

Growing season significantly impacted nearly all nutrient concentrations evaluated in this study (**Tables 3**, **5**). Many of the nutrients were lowest under 6 h LED treatment (**Tables 2**, **4**). Tissue P concentrations were highest in the 12 h LED treatment, with elevated levels shown in most LED treatments and HPS treatments. The NL controls had slightly lower levels of P, but the lowest concentration was observed in the 6 h LED treatment. Tissue Ca concentrations were significantly impacted, with 12 h LED again being the optimal lighting treatment. Levels vary among LED treatments, and separation does not exist between many of the LED treatments and the HPS treatment. The 6 h LED treatment showed lower Ca levels but with varying levels of significance. Tissue S concentrations were highest in the 24 h LED and 6 h HPS treatments, with significance among other LED treatments. The 6 h LED again had the lowest concentration. Basil Mg levels showed some variance across lighting treatments. All micronutrients were impacted by lighting treatment.

The combination of circadian rhythm, temperature, and light-induced stomatal were likely significant factors for unexpected trends in biomass production and nutrient accumulation. Additional experiments are needed to verify this speculation. While temperature variation is known to induce stomatal opening, one important factor that regulates stoma position is blue and red wavelengths (Briggs and Huala, 1999). At low PPFD (15 to 30 μmol·m^-2^·s^-1^), blue light induces stomatal opening, while red light is ineffective; as PPFD increases, stomatal opening is consistently higher for blue light than red light under concurrent PPFD, making the process more sensitive to blue wavelengths (Briggs and Huala, 1999; Olle and Virsile, 2013; Matsuda et al., 2016). In addition, stomatal conductance in leaves subjected to blue and red wavelengths was shown to be higher than in leaves subjected to only blue or red wavelengths, suggesting a synergistic action of stomatal regulation. It is worth noting that this increase in stomatal conductance under blue wavelengths may result from additive or synergistic effects with red light (and other wavelengths) that was possible from morphogenic responses including increased stomatal density, width, and length (Tuan et al., 2013; Chong et al., 2014). Transpiration, gas exchange, and water uptake all influence plant growth and development, in addition to biomass accumulation and nutrient content. Increased transpiration rates allow more nutrients to be absorbed by the plant. Increased water uptake is an important driver of photosynthesis and respiration, both of which are responsible for FM and DM. Higher levels of transpiration (induced by SL lighting) increase the amount of CO_2_ that passes through the stoma, resulting in a higher carbon fixation rate (i.e., photosynthetic rate) and increases in net biomass accumulation. Based on the data and literature currently availability, we believe a combination of these factors resulted in unexpected FM/DM, as well as the nutrient bioaccumulation interactions between treatment and season.

### 4.4 Energy efficiency

Electrical energy, specifically used for SL, is one of the top three most expensive business costs of common commercial greenhouse operations (Singh et al., 2015; Engler and Krarti, 2021). Maximizing efficiency with improved lighting regimes will be vital as global energy demand continues to increase. There is significant potential to utilize continually advancing SL technologies, along with physiology-based application protocols, to develop species-specific lighting regimes for yield and nutrient uptake optimization (Graamans et al., 2018; Jayalath and van Iersel, 2021; Tabbert et al., 2021).

One strategy to improve the efficiency of SL includes applying different spectral qualities of light EOD to extend daylength and increase DLI which, in theory, should improve yields/nutritional quality. **Tables 6**, **7** provide a detailed comparison of how different SL types and applications influenced yields in terms of energy consumption. By comparing the total treatment DLI, total daylength, and fresh yield columns, it is clear that DLI and daylength are not the only drivers of yield (**Tables 6**, **7**). Biomass efficiency (BE), which is a measure of yield per unit of average DLI received by plant throughout lifecycle, was highest for the HPS 6 h and lowest for the LED 9 h treatment. The NL control had the second-highest BE value, likely because the majority of DLI was provided by the natural solar spectrum. Many of the treatments actually reduced the BE, which is a sign that the plants were not able to utilize the provided photons efficiently to produce biomass.

SL energy input per yield increase and total yield increase per SL energy cost values represents the efficiency of additional SL light to yield increases in terms of electrical energy used and price. It was interesting to see that many of the provided treatments were not an efficient use of electricity. In some of the LED treatments, the values were negative, meaning that the energy provided unexpectedly decreased crop yield; in other words, money could have been saved and basil crop yields increased by just using natural sunlight and not applying some SL treatments. This demonstrates that SL applicating timing and spectral quality is a critical factor when considering electrical efficiency. Not all SL treatments are guaranteed to improve yields, and some could even be detrimental to both yields and business operating expense.

Light use efficiency (LUE) is a metric that reflects the total amount of carbon assimilation that occurs during a plant’s life cycle relative to the amount of light intercepted by a plant, typically measured in terms of the ratio of mass to mols of absorbed photosynthetically active radiation (PAR). This metric is essential for understanding the efficiency of light energy conversion into biomass production in plants. The blue (400-500 nm) and red (600-700 nm) regions of the spectrum are particularly important for photosynthesis, as they correspond to the absorption peaks of chlorophyll pigments. However, the green-yellow region (500-600 nm) is less efficiently utilized due to the lower absorbance of chlorophyll in this range, leading to a lower LUE in this spectral region.

This experiment found many of the EOD LED treatments were not an efficient use of electrical energy when compared to the control. Many of the lower-DLI treatments caused significant reductions in yield and LUE, while also consuming electrical energy. We expected that increasing DLI would improving the yield regardless of the lighting type or application timing, but the results show that both application timing and spectral quality of SL are equally as important as DLI and energy efficiency of SL system. Spectral quality of light can also influence LUE by affecting various aspects of plant physiology, such as the rate of photosynthesis, photomorphogenesis, and circadian rhythms. By understanding the relationship between LUE and spectral quality, researchers can optimize lighting systems for plant growth, improve crop yield, and develop more efficient horticultural practices.

As discussed previously, HPS lights emit a broad spectrum of light with a strong emphasis on red and yellow wavelengths, which are beneficial for plant growth during the flowering stage. However, HPS lights have a lower spectral output in the blue wavelength range, which is crucial for vegetative growth. LED lights can be designed to emit specific wavelengths of light tailored to various stages of plant growth. They can also provide a balanced spectrum of blue, red, and far-red light, which can be adjusted according to specific crop needs. The differences in spectral output between HPS and LED lights can affect the photosynthetic efficiency of plants. LED lights can potentially provide a more targeted spectral output that closely aligns with the absorption peaks of chlorophyll a and b, leading to more efficient photosynthesis. This targeted spectral output can also reduce the amount of wasted energy from light that is not absorbed by the plant, increasing overall energy efficiency.

Energy efficiency in horticultural lighting systems is typically measured in terms of photosynthetic photon efficacy (PPE), which is the ratio of the photosynthetically active radiation (PAR) output to the electrical input power (µmol·J^-1^). Due to their targeted spectral output and lower heat production, LED lights generally have a higher PPE than HPS lights. LEDs are inherently more efficient than HPS lamps at converting electrical energy into light energy. LEDs have significant potential for decreasing energy costs for SL while improving yields and nutritional quality, but this study indicates lighting regime is just as important as considering the energy consumption and PPE of any given lighting system. It should be noted that the electrical efficiency of SL systems is highly dependent on a number of other factors that were not discussed or evaluated in this experiment. Some of these factors include growing facility location and setup, lighting fixture type, crop species and variety, optimization of standard controlled environment agriculture parameters (i.e., light, temperature, etc.), plant spacing, adequate ventilation, and fertility regime.

Because of the many environmental factors that influence plant growth and development, it can be inherently difficult to compare similar greenhouse and growth chamber studies involving plant/light interaction. Further evaluation of SL strategies across a variety of specialty crops, specifically the interaction of spectral quality, DLI, and application timing under greenhouse conditions, should be conducted to determine the most energy-efficient methods for increasing yields while balancing other quality parameters like flavor, nutritional quality, and visual appearance. This experiment evaluated one of the most popular herb culinary varieties, but comparative studies using novel varieties of high-value specialty crops would be particularly useful. Photosynthetic efficiency, stomatal conductance, carbon metabolism, and circadian rhythm manipulation using SL should be further explored in terms of energy efficiency and LUE. A thorough understanding of species-specific physiological responses to light will help guide the development of next-generation SL technologies and energy optimization strategies.

### 4.5 Limitations and future considerations

The original aim of this experiment was to investigate the effects of SL application timing, duration, spectral quality on biomass and nutrient uptake across seasons. This was done with the end goal of improving the energy efficiency of SL by optimizing application schedule. We expected to observe a linear increase in biomass with increased SL DLI and some variation across spectral quality. We can accept our initial alternate hypothesis, which was grounded in a comprehensive literature review and established methodologies. However, upon analyzing the results, we were surprised to find each experimental factor (i.e., SL application timing, duration, spectral quality, and seasonal variation in ambient sunlight) differentially influenced biomass, nutrient bioaccumulation, and LUE. These unexpected findings did not align with our original expectations and prompted us to speculate on alternative explanations for the observed outcome. These results establish a proof-of-concept for low-intensity EOD SL, and open up exciting opportunities for future research; specific physiological mechanisms discussed in this manuscript should be replicated and verified in future experiments using further analytical and molecular techniques.

An important factor in primary metabolism and circadian rhythm is plant temperature. While energy costs of heating and cooling were not evaluated in this study, greenhouse temperature maintenance and optimization are vital for successful, efficient, and sustainable crop production. It is well known that LEDs produce significantly less radiant heat energy than HPS lighting systems (Morrow, 2008). Most of the radiant heat energy produced by HPS lamps is directed at the crop canopy’s surface, while LED heat is mostly radiated through thermal sinks and directed away from the canopy’s surface. Consequently, the ambient temperature of the greenhouse and leaf temperature averages may be impacted depending on the design of the heating/cooling systems, outside temperatures, and the distance between the crop and lighting source.

HPS lighting sources contribute more radiant heat energy to the canopy’s surface temperature than LEDs, which may prove useful during cold months by increasing photosynthetic rates as compared to plants without that additional heat. Increased leaf temperatures from infrared (IR) wavelengths will directly increase leaf temperatures as well as metabolic activity and transpiration. While it is more energy-efficiency to raise plant temperatures using typical greenhouse heating systems to raise ambient temperature, in some cases, higher canopy temperatures may prove advantageous for yield optimization. IR wavelengths from HPS sources may conversely prove harmful for plants with low leaf temperature requirements, especially during summer months with high light intensities and ambient temperatures, resulting in heat stress and photodamage. Additional considerations for greenhouse cooling systems and continual adjustments to ambient air temperature may be required to keep canopy temperatures consistent across the day and night periods, requiring additional ventilation strategies or specific on/off schedules.

Excess radiant heat, in the form of infrared (IR) wavelengths, produced from HPS sources during warm growing seasons may explain why changes to FMs and DMs were not as pronounced during these seasons, as heat stress is known to reduce biomass and induce thermomorphogenic responses. It is possible that excess IR wavelengths hindered optimal photosynthetic rates, in addition to variance in day/night temperatures between experimental treatments that may have influenced FM/DM. By extending/increasing night temperatures, HPS treatments may have increased metabolic rates, but not allowed for a cooler rest period overnight, leading to a stagnation in yield between the 6 h and 12 h HPS (**Figure 3A**).

In this experiment, our aim was to minimize air temperature and leaf temperature variation across treatments to primarily focus on the interaction of SL and NL. Leaf temperatures were checked periodically throughout the experiment (during light calibration) to verify treatment environments were similar. HPS lights were hung at a higher distance to reduce the likelihood of increased leaf temperatures. 24 h continuous leaf temperature measurements would have allowed for precise record of any discrepancies among treatments and should be investigated further (i.e., potential for using heat from SL light to increase leaf temps rather than increase air temperature). While we did not investigate air temperature or leaf temperatures under the different SL regimes, it would be valuable to evaluate the influence of temperature microenvironments from SL sources within greenhouse spaces across different seasons.

One limitation of this study is that multiple replications of each growing season were not conducted (i.e., multi-year experiment). Mixed Model ANOVA and GLIMMIX statistical procedures (treatments nested within seasons) indicated season significantly influenced FM, but did not show significant season*treatment interactions (*P*=0.1235; F=9.62). That being said, there are clear visual patterns that suggest season*treatment interactions (**Figures 5**, **6**); a multi-year experiment would provide additional statistical power and likely elucidate any potential significant season*treatment interactions.

Testing different DLIs at constant application durations (i.e., higher SL intensities) to elucidate DLI/lighting period duration effects across growing seasons would be another potential strategy and help isolate the variable of SL intensity; however, it would be difficult under standard greenhouse conditions because the PPDF, DLI, and photoperiod of ambient sunlight are constantly changing, and this type of experiment would ideally be performed in growth chambers. It is extremely difficult to precisely control of DLI from ambient sunlight in greenhouses (i.e., precisely limit ambient sunlight using shade clothes, etc.). That being said, it is very simple to supplement ambient sunlight with SL and is more applicable to commercial growers. Because the ambient sunlight cannot be controlled precisely, we decided to take the approach of not trying to control the sun; rather, control the SL applications and see how they interact with the ambient solar spectrum as it changes over the year. We believe both approaches have merits and should be used together in order to elucidate effects from each of the pertinent lighting parameters.

Irrespective of electrical cost, the FM and DM results of this study suggest narrowband B/R is much better applied during the daytime or full 24 h, while the HPS is better applied at night for 6 h; LED treatment yields began to improve above the control after daylight hours, while the 6 h HPS treatment did not see yield improvements despite adding an additional 6 h of EOD SL. It is likely that the natural solar spectrum was optimized during daylight hours by providing specific narrowband B/R wavelengths, which are primary drivers of photosynthesis and promote several physiological responses. Narrowband wavelengths at night may have boosted yields if other wavebands within the visible spectrum were included (i.e., broadband white with optimized B/R). Further, 18 h and 24 h LED SL may have overridden endogenous circadian rhythm, or the continuous application (no dark period) failed to disrupt the balance of light-dependent and independent reactions significantly. Because photosystems were kept active 24 h per day, light-dependent reactions were able to occur nonstop (all while light-independent reactions were free to occur). On the other hand, maintaining a natural day length (i.e., 9.9 h to 13.5 h) with higher intensities of SL may be useful for synchronous light/dark reactions, and increased biomass accumulation, given that sufficient DLI and spectral quality requirements are met. These results suggest that light schedule and exposure length of SL are equally important as DLI for biomass accumulation. While the intention of this experiment was to investigate the use of SL under commercial greenhouse conditions, multi-year tandem experiments with sole-source lighting in growth chambers would prove useful to further elucidate other spectral quality effects and eliminate confounding factors (i.e., DLI and intensity). Future studies should also consider the development of lighting protocols that are optimized for across different high-value specialty crops that incorporate plants with/without photoperiod requirements during commercial production as well as crops with species-specific tolerance to 24 h lighting periods (i.e., sweet basil does not have a photoperiod requirement when grown to vegetative maturity and can tolerate 24 h of light per day).

In this study, changes to the spectrum, DLI, and application timing of SL lighting differentially regulated the absorption of mineral nutrients. Recent experiments have evaluated the effects of narrowband wavelengths on nutrient uptake with mixed effects, highly dependent on lighting type and species (Kopsell and Sams, 2013; Samuoliene et al., 2013; Pinho et al., 2016; Amoozgar et al., 2017; Samuolienė et al., 2017; Brazaitytė et al., 2018; Pennisi et al., 2019; Hammock et al., 2020; Meng et al., 2020; Brazaityte et al., 2021; Boldt and Altland, 2022; Vaštakaitė-Kairienė et al., 2022a; Vaštakaitė-Kairienė et al., 2022b). The primary focus of this experiment was to evaluate micronutrients, but comparative experiments evaluating all essential nutrients (including N) would be beneficial. Specific PD and EOD lighting regimes should be further explored to determine the interactions between SL spectral quality, DLI, application timing, and their influence on mineral nutrient uptake, assimilation, and bioaccumulation.

Potential advantages for production can be achieved by manipulating PD or EOD light in a controlled environment; the most notable, however, has been achieved through manipulations of photoperiod and/or DLI (Chinchilla et al., 2018). Multiple studies have reported that adding PD-blue and/or EOD-red light can efficiently increase the yield of plants (Sung and Takano, 1997; Hanyu and Shoji, 2002; Fraszczak, 2013; Jishi et al., 2016; Kuno et al., 2017), which coincide with the results of this experiment (where applicable). The results of these studies demonstrate that the quality of the SL lighting source has a direct impact on yield, likely caused by several physiological responses elicited from specific narrowband wavelengths within SL light treatments. Further metabolomic and transcriptomic studies are needed to determine the precise mechanisms behind the yield differences observed in this study and to improve our understanding of plant/light interaction. Innate limitations posed by greenhouse SL and growth chamber sole-source lighting experiments can be overcome by executing parallel experiments and comparing a variety of growth, developmental, and physiological parameters. Additionally, three “circadian rhythm-based” lighting strategies should be further explored: 1) complete synchronization of SL with a plant’s endogenous circadian clock and natural sunlight to optimize all physiological processes and yield; 2) minimally disruptive SL treatments to extend natural daylength by providing discrete wavelengths at certain times throughout the day; and 3) constructively disruptive SL treatments, relative to natural sunlight, that would force the manipulation of endogenous circadian rhythm and other metabolic processes to promote certain desirable traits (i.e., yield, changes to resource allocation, carbon metabolism, flavor, and morphology).

## 5 Conclusions

The results of this experiment clearly indicate that SL spectral quality, DLI, and application timing greatly influence fresh/dry yields, nutrient uptake, and SL energy efficiency in greenhouse hydroponic basil cultivation. Across all seasons, HPS lights provided optimal results for both fresh/dry edible biomass yield (25-29% over control), while 9 h to 18 h LED treatments significantly increased the uptake of many macro- and micronutrients when compared to HPS and natural light controls.

For all parameters considered in this study, the optimal supplemental DLI ranged from 4.2-6.3 mol·d^-1^. Lighting type (i.e., spectral quality) and application timing were significant factors in terms of biomass accumulation, nutrient uptake, and electrical efficiency. In general, PD-blue and EOD-red wavelengths were beneficial for yield increases. SL treatments had different effects across growing seasons, due to changes in the spectral quality, DLI, and daylength of ambient sunlight.

In our efficacy comparison, LEDs and HPS have proven merits, but both systems have limitations in regard to nutrient cycling and sustainability under controlled environments. Biomass efficiency across treatments varied two-fold. When considering SL energy costs, LED 18 h was the most cost-effective treatment for increasing mass. Additional efficacy comparisons between HPS and LED lighting systems should be conducted on a variety of physiological metrics to determine economically favorable practices. Plant quality and other secondary metabolic interactions were not evaluated in this experiment and should be considered for future experiments; while increased yields are important, secondary metabolites and quality attributes are also important for consumer acceptance and preference. Using metabolomic and transcriptomic methodologies, future studies should investigate these types of lighting regimes to determine responsible physiological and biochemical mechanisms and how those can be leveraged to increase yields and nutritional quality of specialty crops grown in controlled environment agriculture systems.

## Data availability statement

The raw data supporting the conclusions of this article will be made available by the authors, without undue reservation.

## Author contributions

Conceptualization – HH, DK, CS. Methodology – HH, CS. Software – HH, CS. Validation – HH, CS. Formal analysis – HH, CS. Investigation – HH, CS. Resources – CS. Data curation – HH. Writing (original draft preparation) – HH. Writing (review and editing) – HH, DK, CS. Visualization – HH. Supervision and project administration – CS. Funding acquisition – CS. All authors contributed to the article and approved the submitted version.
